# CRISPR-Cas System in Antibiotic Resistance Plasmids in *Klebsiella pneumoniae*

**DOI:** 10.3389/fmicb.2019.02934

**Published:** 2020-01-10

**Authors:** Muhammad Kamruzzaman, Jonathan R. Iredell

**Affiliations:** ^1^Centre for Infectious Diseases and Microbiology, The Westmead Institute for Medical Research, The University of Sydney, Westmead, NSW, Australia; ^2^Westmead Hospital, Westmead, NSW, Australia

**Keywords:** *Klebsiella pneumoniae*, *Enterobacteriaceae*, plasmid, CRISPR, antibiotic resistance

## Abstract

CRISPR-Cas (clustered regularly interspersed short palindromic repeats-CRISPR-associated protein) is a microbial adaptive immune system involved in defense against different types of mobile genetic elements. CRISPR-Cas systems are usually found in bacterial and archaeal chromosomes but have also been reported in bacteriophage genomes and in a few mega-plasmids. *Klebsiella pneumoniae* is an important member of the Enterobacteriaceae with which they share a huge pool of antibiotic resistance genes, mostly via plasmids. CRISPR-Cas systems have been identified in *K. pneumoniae* chromosomes, but relatively little is known of CRISPR-Cas in the plasmids resident in this species. In this study, we searched for CRISPR-Cas system in 699 complete plasmid sequences (>50-kb) and 217 complete chromosomal sequences of *K. pneumoniae* from GenBank and analyzed the CRISPR-Cas systems and CRISPR spacers found in plasmids and chromosomes. We found a putative CRISPR-Cas system in the 44 plasmids from *Klebsiella* species and GenBank search also identified the identical system in three plasmids from other Enterobacteriaceae, with CRISPR spacers targeting different plasmid and chromosome sequences. 45 of 47 plasmids with putative type IV CRISPR had IncFIB replicon and 36 of them had an additional IncHI1B replicon. All plasmids except two are very large (>200 kb) and half of them carried multiple antibiotic resistance genes including *bla*_*CTX–M*_, *bla*_*NDM*_, *bla*_*OXA*_. To our knowledge, this is the first report of multi drug resistance plasmids from Enterobacteriaceae with their own CRISPR-Cas system and it is possible that the plasmid type IV CRISPR may depend on the chromosomal type I-E CRISPRs for their competence. Both chromosomal and plasmid CRISPRs target a large variety of plasmids from this species, further suggesting key roles in the epidemiology of large plasmids.

## Introduction

Acquisition of genetic material including virulence, fitness and antibiotic resistance genes by horizontal gene transfer (HGT) is an essential process in bacterial adaptation to different environments (Frost et al., 2005). In addition bacteria have acquired an adaptive immune system, clustered regularly interspaced short palindromic repeats and their associated Cas proteins (CRISPR-Cas), which helps to limit the acquisition of genetic materials and defend against invasive bacteriophages and plasmids (Garneau et al., 2010; Barrangou, 2015; Samson et al., 2015).

A typical CRISPR-Cas locus is comprised of a CRISPR array, Cas genes and a leader sequence. A CRISPR array is comprised of nearly identical short (21 to 47 nucleotides) direct repeats, separated by unique DNA fragments (spacers) acquired from foreign DNA [mobile genetic elements (MGEs)]. The leader sequence is usually a (∼100–500 bp) AT rich region believed to serve as a promoter for the transcription of the CRISPR array (Marraffini, 2015). The CRISPR-Cas defense mechanism can be considered as three steps. In an initial adaptation step foreign DNA fragments (protospacers) from infecting bacteriophages and plasmids are incorporated into the CRISPR array as new spacers. These spacers provide the sequence specific memory for a targeted defense against subsequent invasions by the same bacteriophage or plasmid. The CRISPR array transcript is then processed to matured CRISPR RNAs (crRNAs). After expression of the array, mature crRNAs, aided by Cas proteins, identify specific targets and cleave the nucleic acid strands of corresponding viruses or plasmids (van der Oost et al., 2009; Garneau et al., 2010; Makarova et al., 2011b, 2015; Barrangou, 2015).

CRISPR-Cas systems show a great deal of diversity in their Cas protein composition, structure of effector proteins complex, genetic organization and localization in the genome, mechanism of adaptation, crRNA processing and interference. Based on the effector complexes CRISPR-Cas systems can be divided into two classes and six types (Class 1, including types I, III, IV and class 2, including types II, V and VI), those can be sub-divided into at least 34 sub-types (Makarova et al., 2011b, 2015, 2018; Koonin et al., 2017; Hille et al., 2018), Class 1 CRISPR-Cas system provides interference by using multi-Cas effector protein complex whereas Class 2 uses single effector protein for interference (Hille et al., 2018). Generally there are signature genes for each type of CRISPR-Cas system and those include *cas3* for type I, *cas9* for type II, *cas10* for type III, *csf1* (large subunit, *cas8-*like) for type IV, *cas12* for type V, and *cas13* for type VI (Makarova et al., 2018). Types I and II CRISPR-Cas systems provide immunity against DNA (Brouns et al., 2008; Gasiunas et al., 2012) whereas type III systems may target DNA or RNA (Tamulaitis et al., 2014). Types I–III are well-studied and are generally found in chromosomes of bacteria and archaea, whereas types IV, V, and VI are three putative new types. Type IV systems are usually localized on plasmids or other MGEs and lack apparent adaptation modules (*cas1* and *cas2*) and type V was identified in archaeal chromosome only (Makarova et al., 2011a, 2015). Type VI is another new type identified recently carrying HEPN-domain containing effector protein Cas13, which, unlike, other class II effector cleaves single stranded RNA (ssRNA) (Hille et al., 2018). HEPN RNase is a toxin domain of bacterial toxin-antitoxin module and suggests that type VI includes dedicated RNA-targeting CRISPR-Cas system (Makarova et al., 2011b, 2018; Koonin et al., 2017).

The classification, functions and mechanism of actions of all CRISPR-Cas systems are well-characterized except type IV. A recent study demonstrated that the function of type IV system in the maturation of crRNAs and in the subsequent formation of a Cascade-like crRNA-guided effector complex (Ozcan et al., 2019). Type IV system can be classified as two sub-types, type IV-A and IV-B, based on the presence of DinG family helicase and type IV specific effector protein Csf5. Type IV-A encodes a DinG helicase (Csf4) and an effector protein Csf5 and whereas type IV-B lacks these proteins (Makarova et al., 2015, 2018; Koonin et al., 2017; Hille et al., 2018; Pinilla-Redondo et al., 2019) and usually, the type IV-A system carries CRISPR-array.

*Klebsiella pneumoniae*, a member of the bacterial family Enterobacteriaceae, is a common opportunistic hospital associated pathogen, accounting for about one third of total Gram-negative infections (Navon-Venezia et al., 2017). It causes a variety of infections including urinary tract infections, pneumonia, cystitis, wound infections, and life-threatening sepsis (Podschun and Ullmann, 1998). Occurrence of transmissible antibiotic resistance in this organism is a major problem worldwide. *K. pneumoniae* have a huge pool of antibiotic resistance genes that they share among other Enterobacteriaceae, mostly via self-transferrable plasmids (Navon-Venezia et al., 2017). Almost all modern antibiotic resistance (to carbapenems, cephalosporins, aminoglycosides, now even colistin) in these organisms is encoded on large (40–200 kb) low-copy (1–6 per cell) conjugative plasmids (Carattoli, 2009; Navon-Venezia et al., 2017). Plasmid-borne antibiotic resistance is acquired very quickly and, once acquired, could become fixed in the bacterial accessory genome by ‘addiction systems’ that poison cells from which the antibiotic resistance plasmid is lost (Hayes, 2003).

Several studies have identified CRISPR-Cas systems in *K. pneumoniae* chromosomes as I-E and I-E^∗^ types (Ostria-Hernandez et al., 2015; Shen et al., 2017; Li et al., 2018) but little is known about CRISPR-Cas systems of plasmids in *K. pneumoniae* and other Enterobacteriaceae (Enas Newire et al., 2019). CRISPR-Cas systems are associated with relative antibiotic susceptibility in *Streptococcus pyogenes* and *E. coli* and the chromosomal CRISPR-Cas system is known to interfere with acquisition of antibiotic-resistant plasmids in *E. coli* (Zheng et al., 2014; Aydin et al., 2017). In this study, we examined 699 complete plasmid sequences from *K. pneumoniae* and 217 *K. pneumoniae* chromosomal sequences from the GenBank for the presence of CRISPR-Cas system and further analyzed the identified CRISPR-Cas systems and their spacers.

## Materials and Methods

### Extraction of Complete Nucleotide Sequence of Plasmids and Chromosomes for *K. pneumoniae* From the GenBank

*Klebsiella pneumoniae* chromosome and plasmid sequences available in the GenBank database^1^ were downloaded and subjected to CRISPR analysis. For complete *K. pneumoniae* chromosomal sequences, after opening the database link, we selected “genome assembly and annotation report” and chose complete sequences and then extracted all the complete nucleotide sequences individually and saved as FASTA format sequence file. For complete plasmid sequences, in the same link we selected “plasmid annotation report” and downloaded plasmid sequences > 50 kb and saved as separate FASTA files for individual plasmid sequences.

### Identification and Characterization of CRISPR-Cas in Plasmid and Chromosomal Sequences

CRISPR was identified with CRISPRFinder^2^ (Grissa et al., 2007) software. This algorithm locates direct repeat sequences of 23–55 bp separated by variable sequences of a size no greater than 2.5 times or no less than 0.6 times the length of the repeated sequences (25–60 bp). When the algorithm detects at least three repeating regions that are exactly the same (in sequence and size), which are separated by variable sequences, it is considered a “confirmed CRISPR.” If the algorithm locates two repeats separated by a variable sequence, it establishes the status of a “questionable CRISPR.” For the present study we only considered those indicated by the program as “confirmed CRISPRs.” In addition, with this platform, we searched for *cas* genes in regions adjacent to CRISPR sequences. Fasta formatted complete nucleotide sequence of each individual plasmid or chromosome was uploaded in the CRISPRFinder and run the program by using a default setting parameters and outcomes provided the possible CRISPR-array (CRISPR repeats and spacers). Spacers sequences were collected from CRISPRFinder outputs and saved to use for further analysis. The CRISPR region identified by CRISPRFinder was then detected on the plasmid or chromosomal sequences and nearly 10 kb upstream and downstream regions were analyzed for putative *cas* genes. The CRISPR-array neighboring genes and their respective protein sequences were analyzed by BLASTn and BLASTp searches for the GenBank identity. For nucleotide sequence analysis, megablast was performed by using following parameters: (i) expectation threshold (e-values) less than or equal to 0.01 and a score greater than 40, (ii) maximum target sequence was set at 1000, (iii) automatically adjusted parameters for short input sequences, (iv) different match/mismatch scores were selected to identify highly conserved to low conserved sequences. BLASTp for protein sequences were performed against non-redundant protein sequence database and against reference proteins sequence database with expectation values (e-value) less than or equal to 0.01 were considered significant as well as a coverage percentage of more than or equal to 80%. The identified CRISPR-array and *cas* genes were further verified by using CRISPRone software^3^ (Zhang and Ye, 2017). The individual fasta formatted nucleotide sequence of plasmid and chromosome was run through CRISPRone software by using default settings.

### Search for Similar CRISPR-Cas System in GenBank Data

The *cas* genes identified in the putative type IV CRISPR-Cas systems in the plasmids of *K. pneumoniae* were used to fish similar type of CRISPR-Cas system in the GenBank data. Both the *cas* genes nucleotide sequences and amino acid sequences were used separately for BLASTn and BLASTp search in the GenBank data with the parameters mentioned earlier. The additional plasmid sequences identified with identical *cas* genes or Cas proteins were downloaded and analyzed for CRISPR-array and *cas* genes orientation by CRISPRFinder and CRISPRone software.

### Analysis of CRISPR Spacers and Identification of Spacers Protospacers Match

Spacers from respected plasmid CRISPR-Cas system were extracted from CRISPRFinder outputs and made a fasta formatted sequence file for all spacer pool by BioEdit software^4^. Each of the spacers sequence, their reverse complement sequence and both 3′ and 5′ truncated version were then searched against the spacer pool and identified all the unique spacers found in the plasmid CRISPRs and then plotted their distribution. Each of the unique spacer was then analyzed for their identity (match with protospacers) to GenBank sequences by nucleotide blast search (BLASTn) with parameters described earlier.

### Identification of Chromosomal CRISPR Type

Two different types of *cas1* and *cas3* alleles were found in *K. pneumoniae* genomes and CRISPR-Cas systems were further divided into types I-E or I-E^∗^ (Li et al., 2018) on the basis of the *cas1* and *cas3* alleles and their localization in the chromosome.

### Plasmid Characterization

The presence of antibiotic resistance genes in sequenced plasmids were identified by ResFinder 3.2^5^ and plasmid replicon types by PlasmidFinder 2.1^6^ (Carattoli et al., 2014).

## Results

### CRISPR-Cas System in *K. pneumoniae* Plasmids

**FIGURE 1:**
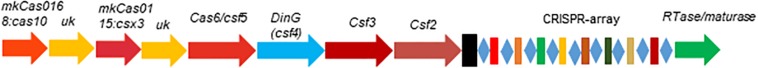
Schematic representation of type IV-like CRISPR-Cas system in plasmids found in Enterobacteriaceae. Putative genes are identified. CRISPR associated genes *(Cas)* are shown in red, *DinG helicase* in blue, unknown/hypothetical genes in yellow. Black rectangle indicates a putative leader sequence; blue diamonds are for CRISPR-repeats and colored rectangles are for acquired spacers, together consisted CRISPR-array.

A total of 699 complete plasmid sequences of > 50-kb in size found in *K. pneumoniae* were extracted from the GenBank database. CRISPR-arrays were identified in 5% (37 of 699; Table 1 and Supplementary Table S1) of the plasmids. The identified CRIPSR-arrays had direct repeats of 23–30 bp separated by a variable number (0–22) of spacer sequences of 25–57 bp and most of them are 30–33 bp long (Supplementary Table S2). Immediately upstream of the CRISPR-array an ∼130 bp conserved AT rich region was present, which may act as a leader sequence of this CRISPR. We also identified *csf2*, *csf3*, *DinG helicase (csf4)*, *cas6* (*csf5*), *csx3*, and *cas10* homologs upstream and a reverse transcriptase (*RTase*) or maturase gene downstream of the CRISPR-array (Figure 1). Two genes of unknown function were also present in the *Cas* genes locus, but we could not identify the adaptation genes *cas1* or *cas2*, or evident homologs, in these plasmids. The structure of the CRISPR-Cas array and organization of *cas* genes identified here is very close to that of the type IV CRISPR-Cas system previously identified in the mega-plasmid of *Aromatoleum aromaticum* EbN1, an aromatic-degrading betaproteobacteria found in freshwater and soil habitats (Ozcan et al., 2019), even though a large subunit (Csf1) that acts as the signature protein for this type (Makarova et al., 2015) is absent from the system identified here. The *Cas* genes and their orientations in identified CRISPR-Cas systems are very similar among the plasmids except for the presence of Insertion Sequences (ISs) insertion events in some. ISs were also identified between the CRISPR-array and *RTase* gene in a few plasmids.

**TABLE 1:** Characteristics of CRISPR-Cas positive plasmids.

**Sl no.**	**Plasmid**	**Species^a^**	**Accession no.**	**CRISPR-array position**	**DR-length (bp)**	**No. of spacers**	**Plasmid size (kb)**	**Replicon type^b^**	**Antibiotic resistance genes identified (annotation by ResFinder^c^)**
1	pKPM501	*Kp*	CP031735.1	200609–201185	28	9	253	IncFIB_*k*_, IncFII_*k*_	*bla*_*CTX–M–*__15_, *bla*_*TEM–*__1__*A*_, *aac(6′)-Ib, aac(6′)-Ib-cr, dfrA14*
2	unnamed_1	*Kp*	CP022612.1	313185–313943	29	12	335	IncHI1, IncHI1B, IncFIB, IncR	*bla*_*NDM–*__1_, *bla*_*CTX–M–*__15_, *bla*_*TEM–*__1__*A*_, *bla*_*OXA–*__9_, *bla*_*SHV–*__13_, *aph(3′)-VI, armA, aadA2, aadA1, aac(6′)-Ib, aac(6′)-Ib-cr, aac(3)-IId, aph(6)-Id, sul1,2,3; dfrA12*
3	AR_0153 plasmid unnamed1	*Kp*	CP028929.1	62241–63001	29	12	283	IncHI1B, IncFIB	*bla*_*NDM–*__1_, *bla*_*OXA–*__1_, *aph(3′)-VI, armA, aadA2, aac(6′)-Ib-cr, qnrB1, sul1, dfrA12, dfrA14*
4	pKPN528-1	*Kp*	CP020854.1	251737–252495	29	12	292	IncHI1B, IncFIB	*bla*_*NDM–*__1_, *bla*_*OXA–*__1_, *aph(3′)-VI, armA, aadA2, aac(6′)-Ib-cr, qnrB1, sul1, dfrA12, dfrA14*
5	AR_0068 plasmid unitig_1	*Kp*	CP020068.1	136244–137004	29	12	276	IncHI1B, IncFIB	*bla*_*NDM–*__1_, *bla*_*SHV–*__13_, *aph(3′)-VI, armA, aadA2, aac(3)-IId, aph(6)-Id, sul1,2; dfrA12*
6	pIncHI1B_DHQP1300920	*Kp*	CP016921.1	229597–230357	29	12	283	IncHI1B, IncFIB	*bla*_*NDM–*__1_, *bla*_*OXA–*__1_, *aph(3′)-VI, armA, aadA2, aac(6′)-Ib-cr, qnrB1, sul1, dfrA12, dfrA14*
7	KP617 plasmid KP-plasmid1	*Kp*	CP012754.1	114404–115164	29	12	273	IncHI1B, IncFIB	*bla*_*NDM–*__1_, *aph(3′)-VI, armA, aadA2, qnrB1, sul1, dfrA12*
8	PittNDM01 plasmid1	*Kp*	CP006799.1	114132–114892	29	12	283	IncHI1B, IncFIB	*bla*_*NDM–*__1_, *bla*_*OXA–*__1_, *aph(3′)-VI, armA, aadA2, aac(6′)-Ib-cr, qnrB1, sul1, dfrA12, dfrA14*
9	pKJNM10C3.2	*Kp*	CP030878.1	136256–137016	29	12	276	IncHI1B, IncFIB	*bla*_*NDM–*__7_, *aph(3′)-VI, armA, aadA2, aph(3′′)-Ib, aph(6)-Id, aac(3)-IIa, sul1, 2; dfrA12*
10	p18ES-342	*Kp*	CM008881	52979–53610	23	10	332	IncHI1B, IncFIB	*dfrA1*
11	unnamed1	*Kp*	CP031818	223392–224022	23	10	430	IncFIB	None
12	pKpvST147L	*Kp*	CM007852	167552–168555	29	16	343	IncHI1B, IncFIB	*armA, aph(3′)-Ia, sul1, 2; dfrA5*
13	KSB2_1B plasmid unnamed1	*Kp*	CP024507.1	41987–42747	29	12	310	IncFIB	None
14	pKp_Goe_414-1	*Kp*	CP018339.1	75291–76111	29	13	204	IncFIB	None
15	pKPN-3967	*Kp*	CP026186.1	221215–221302	27	1	373	IncFIB	None
16	p44-1	*Kp*	CP025462.1	42604–43120	29	8	261	IncFIB	None
17	TVGHCRE225 plasmid unnamed1	*Kp*	CP023723.1	10881–11763	29	14	297	IncHI1B, IncFIB	None
18	pOXA1_020030	*Kp*	CP028791.1	30205–30843	29	10	288	IncFIB	*bla*_*OXA–*__1_, *aac(6′)-Ib-cr, sul1*
19	825795-1 plasmid unnamed1	*Kp*	CP017986.1	148524–149585	30	17	244	IncHI1B, IncFIB	None
20	pKp_Goe_304-1	*Kp*	CP018720.1	24563–25679	24	18	246	IncHI1B, IncFIB	None
21	pKp_Goe_021-1	*Kp*	CP018714.1	7697–8748	30	17	246	IncHI1B, IncFIB	None
22	pKp_Goe_026-1	*Kp*	CP018708.1	54661–55777	24	18	246	IncHI1B, IncFIB	None
23	pKp_Goe_024-1	*Kp*	CP018702.1	5006–6122	24	18	246	IncHI1B, IncFIB	None
24	pKp_Goe_832-1	*Kp*	CP018696.1	102143–103259	24	18	246	IncHI1B, IncFIB	None
25	pKp_Goe_473-1	*Kp*	CP018687.1	116666–117727	30	17	246	IncHI1B, IncFIB	None
26	pKp_Goe_579-1	*Kp*	CP018313.1	94773–95834	30	17	246	IncHI1B, IncFIB	None
27	AR_0363 plasmid unnamed4	*Kp*	CP027156.1	127062–128305	29	20	186	IncHI1B, IncFIB	*aac(6′)-Ib, aac(6′)-Ib-cr*
28	pKPN-edaa	*Kp*	CP026398.1	7401–8525	29	18	249	IncHI1B, IncFIB	*bla*_*NDM–*__1_, *bla*_*OXA–*__1_, *bla*_*DHA–*__1_, *aph(3′′)-Ib, aph(6)-Id, aac(6′)-Ib-cr, qnrB4, sul1*
29	pKPN-bbef	*Kp*	CP026172.1	227283–228407	29	18	244	IncHI1B, IncFIB	None
30	pKpvST101_5	*Kp*	CP031372.1	144828–145262	25	10	210	IncHI1B, IncFIB	None
31	pKpn23412-362	*Kp*	CP011314.1	141865–142618	23	12	362	IncHI1B, IncFIB	*bla*_*OXA*__–__1_, *bla*_*CTX–M–*__15_, *bla*_*TEM–*__1__*B*_, *bla*_*OXA–*__1_, *aac(3)-IIa, aph(3′′)-Ib, aph(6)-Id, aac(6′)-Ib-cr, sul2, dfr1*
32	p1502320-3	*Kp*	CP031580.1	7608–8781	29	19	87	ND	None
33	pKP3301 DNA	*Kp*	AP018748.1	3677–4373	29	11	296	IncHI1B, IncFIB	*bla*_*NDM–*__1_, *bla*_*CTX–M–*__15_, *bla*_*TEM–*__1__*A*_, *bla*_*OXA–*__1_, *aph(3′)-VI, armA, aadA2, aac(6′)-Ib, aac(6′)-Ib-cr*
34	SKGH01 plasmid unnamed 1	*Kp*	CP015501.1	7545–7875	29	15	281	IncHI1B, IncFIB	None
35	Plasmid_A_Kpneumoniae_MS6671	*Kp*	LN824134.1	16764–17823	29	17	280	IncHI1B, IncFIB	None
36	pKJNM8C2.1	*Kp*	CP030858.1	10639–11155	30	8	304	IncHI1B, IncFIB	*bla*_*NDM–*__1_, *bla*_*CTX–M–*__15_, *bla*_*OXA–*__1_, *armA, aadA2, aac(6′)-Ib-cr, qnrB1, sul1, dfrA12*
37	pPMK1-NDM	*Kp*	CP008933.1	10628–11144	30	8	304	IncHI1B, IncFIB	*bla*_*NDM–*__1_, *bla*_*CTX–M–*__15_, *bla*_*OXA–*__1_, *armA, aadA2, aac(6′)-Ib-cr, qnrB1, sul1, dfrA12*
38	pFB2.1	*Pg*	CP014776.1	56849–57852	29	16	242	IncFIB	None
39	pE20-HI3	*Kp*	MG288682.1	27978–29043	29	17	240	IncFIB	*bla*_*OXA–*__1_, *armA, aac(6′)-Ib-cr, sul1*
40	pEC422_1	*Ec*	CP018961.1	276922–277868	29	15	290	IncHI1B, IncFIB	*bla*_*CTX–M–*__2_, *bla*_*TEM–*__1__*B*_, *bla*_*OXA–*__1_, *aac(6′)-Ib-cr, aac(3)-IIa, sul1*
41	pNDM-TJ03	*Kp*	MG845201.1	130378–131435	29	17	280	IncHI1B, IncFIB	*bla*_*NDM–*__1_, *bla*_*OXA–*__1_, *bla*_*SHV–*__12_, *bla*_*DHA–*__1_, *aph(3′′)-Ib, aph(6)-Id, aac(6′)-Ib-cr, qnrB4, sul1*
42	pNDM-TJ11	*Ko*	MG845200.1	130294–131351	29	17	275	IncHI1B, IncFIB	*bla*_*NDM–*__1_, *bla*_*OXA–*__1_, *bla*_*DHA–*__1_, *aph(3′′)-Ib, aph(6)-Id, aac(6′)-Ib-cr, qnrB4, sul1*
43	pENVA	*Kp*	HG918041.1	99454–100629	23	19	254	IncHI1B, IncFIB	*bla*_*NDM–*__1_, *bla*_*OXA–*__1_, *bla*_*SHV–*__12_, *bla*_*DHA–*__1_, *aph(3′′)-Ib, aph(6)-Id, aac(6′)-Ib-cr, qnrB4, sul1*
44	pKP64477b	*Kp*	MF150122.1	84064–85428	29	22	205	IncHI1B, IncFIB	None
45	*Raoultella ornithinolytica* strain 18 plasmid 1	*Ro*	CP012556.1	115417–115897	29	7	216	ND	None
46	pKPN1481-1	*Kv*	CP020848.1	325794–326308	29	8	347	IncHI1B, IncFIB	*bla*_*NDM–*__1_, *bla*_*CTX–M–*__15_, *bla*_*OXA–*__1_, *bla*_*TEM–*__1__*A*_, *bla*_*OXA–*__9_, *aac(6′)-Ib, aac(6′)-Ib-cr, aadA1, qnrB1*
47	pK66-45-1	*Kp*	CP020902.1	None	0	0	338	IncFIB, IncFII, IncHI1B, IncR	*bla*_*NDM–*__1_, *bla*_*CTX–M–*__15_, *armA, aadA2, aph(3′)-VI, qnrS1, sul1, dfrA12*

*^*a*^*Kp, K. pneumoniae; Pg, Pluralibacter gergoviae; Ec, E. coli; Ko, Klebsiella oxytoca; Ro, Raoultella ornithinolytica; Kv, Klebsiella variicola.*^*b*^ND, not detected.^*c*^*aac(6′)-Ib* also commonly annotated as *aacA4; aph(3′′)-Ib, aph(6)-Id* also commonly annotated as *strA, strB.**

### CRISPR-Cas System in Other Enterobacteriaceae Plasmids

A BLAST search identified identical CRISPR-Cas systems in 10 other plasmids in GenBank, in addition to the 37 plasmids identified in *K. pneumoniae* (Table 1). Five of these 10 plasmids were from *K. pneumoniae* and 1 each from *E. coli*, *K. oxytoca*, *Pluralibacter gergoviae, K. variicola*, and *Raoultella ornithinolytica*. One of the plasmids from *K. pneumoniae* has a *cas* locus without any CRISPR-array identified. No match was found in chromosomal sequences on GenBank.

### Characterization of CRISPR-Positive Plasmids

A total of 47 type IV CRISPR-positive plasmids were analyzed. All but two are very large (>200 kb) and the largest is 430 kb (Table 1). Almost all had an IncFIB replicon identified by PlasmidFinder except for two in which no replicon match was identified. Most plasmids (36/47) also have an IncHI1B replicon (Table 1). Interestingly, although most of the plasmids (44/47) were found in *Klebsiella* species, only one plasmid has the characteristic *Klebsiella* type IncFIB_*K*_ replicon (Garcia-Fernandez et al., 2012). The %GC content of almost all CRISPR-negative plasmids is > 50% and lower (∼44–46%) in CRISPR-positive plasmids (Supplementary Table S1). The only CRISPR-positive plasmid with an IncFIB_*K*_ replicon had a GC content of ∼52%, similar to other CRISPR-negative plasmids of *K. pneumoniae*.

ResFinder identified multiple antibiotic resistance genes including *bla*_*CTX–M*_, *bla*_*NDM*_, *bla*_*OXA*_, *armA* and *qnr* genes, associated with resistance to β-lactam, carbapenem, aminoglycoside and quinolone antibiotics, in almost half the plasmids (24/47; Table 1).

### Analysis of Spacers From Plasmid CRISPRs

**FIGURE 2:**
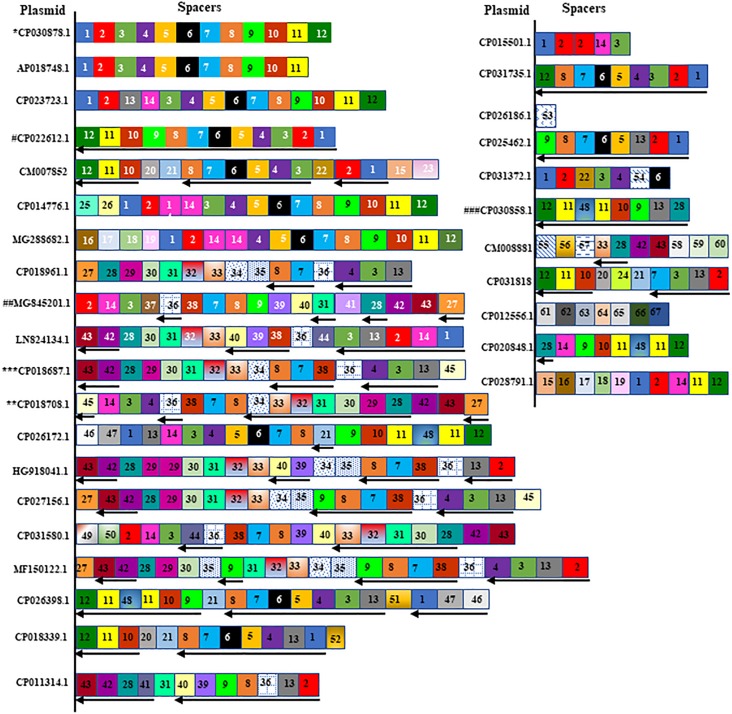
A spacer map for the distribution of spacers in plasmid CRISPRs. Spacers are represented in box without repeats. Identical spacers are represented by same number and color or pattern. Spacers found in reverse orientation in the plasmid CRISPR are shown by reverse arrow at the bottom of the spacer. Exactly same spacers and their orientation are shared by a number of plasmids and are mentioned below in the brackets, and spacers from one of them were represented in the figure. Those plasmids are (^∗^CP030878.1, CP020068.1, CP0016921.1, CP028929.1, CP024507.1, CP020854.1), (^∗∗^CP018708.1, CP018702.1, CP018696.1, CP018720.1), (^∗∗∗^CP018687.1, CP018714.1, CP017986.1), (#CP022612.1, CP012754.1, CP006799.1), (##MG845201.1, MG845200.1) and (###CP030858.1, CP008933.1) and marked with asterisk or hashtag were the representative from each group showed in the figure. The unique spacer sequences and their match with protospacers will be found in Supplementary Table S2.

A total of 623 spacers from 46 CRISPR-positive plasmids were analyzed by BLASTn search, identifying 67 unique spacer sequences that made up the total pool of 623, including some repetition or reversed orientation of the same sequence, along with loss or gain of a few nucleotides (Figure 2 and Supplementary Table S2). Spacer numbers varies without any relationship with plasmid size or the presence or absence of antibiotic resistance genes (Table 1). Five unique spacers (SP11, 36, 43, 55, 57) were found specific for plasmid sequences other than the match with CRISPR-array region and one of them (SP11) appears to match with the *traL* gene of 457 different CRISPR-negative plasmid sequences and another one (SP43) hits the *traN* gene of 260 different CRISPR-negative plasmid sequences in GenBank, mostly (>98%) from *K. pneumoniae* (Table 2 and Supplementary Table S2). Both spacers are found in the CRISPR-array of 17 of the 46 CRISPR-positive plasmids we examined (Table 2). Spacers SP36, SP55, and SP57 have identity with plasmid *transposases*, *transcriptional regulator* and *traH* gene, respectively. Genes, *traL, traN*, and *traH* play an important role in plasmid transfer and are highly conserved among plasmids. Five unique spacers (SP8, 20, 42, 62, 63) were also found to match *K. pneumoniae* chromosomes but not those of their current host bacteria (Table 2 and Supplementary Table S2). Two of them occurred in 17 of the 46 plasmids, targeting *DUF1367 family protein* and *hypothetical protein* genes for 111 and 139 *K. pneumoniae* chromosomes, respectively. Another spacer, that occurred in one plasmid only, appears to recognize a *hypothetical protein* gene from 3 of the *K. pneumoniae* chromosomes (Table 2). Many spacers (18/67) did not have any match in GenBank (Supplementary Table S2).

**TABLE 2:** Spacers from plasmid mediated CRISPR specific to plasmids and *K. pneumoniae* chromosomes.

**Spacer**	**Hits to**	**Target gene**	**No. of occurrence**
CCGAGATTGAGTAAAGCAAAGTAACGGCGGTG	111 *Kp* strains chromosome	*DUF1367* family protein	17
TTCCGGACTCCTGTTTCCGGCAGTGGATTAAA	457 CRISPR-negative plasmids	*traL*	17
CCGAGCTACCGATTTACCAGGAGAGCGCTCGC	139 *Kp* strains Chromosome	*hp*	17
CCGGTTCGGATTTTGCGAAACAGGTGCAGGGC	260 CRISPR-negative plasmids	*traN*	17
TTGGCGACCACCAGCGTTTTAGTGCAGGGAAC	3 *Kp* strains chromosome	*hp*	1
AAGTTATTCATGTCGCCATTCACGTCGGCGGCGTATTT	62 CRISPR-negative plasmids	*traH*	1
TTCTCTCCGCCGGGCAGTGTGATGCCGGAGGGGTATTC	7 CRISPR-negative plasmids	*transcriptional regulator*	1
ATTTACAAATGAAGATTTTTCCCCATTGGTAA	48 CRISPR-negative plasmids	IS200/IS605 family transposase	10
TTCCCTGCACTAAGACGCTGGTGGTCGCCAC	17 Kp strains chromosome	*hp*	9
AGTTTGTATGAAAGCCTCATGTTTTGCACCTGTGCCGG TGCATATCATCCTCAGAGC	6 *Klebsiella* chromosome	*hp*	1

**hp*, hypothetical protein.*

### Analysis of CRISPR-Cas System in *K. pneumoniae* Chromosomes

A total of 217 *K. pneumoniae* complete chromosomal sequences were extracted from GenBank (June 2019) and we identified that 81 of these (37%) carried CRISPR-Cas system on the chromosome. Of these 81, 45 were I-E and 36 were I-E^∗^ type (Supplementary Table S3), consistent with previous reports (Ostria-Hernandez et al., 2015; Shen et al., 2017; Li et al., 2018).

### Relationship Between the Presence of Chromosomal CRISPR and Plasmid in *K. pneumoniae* Bacteria

**FIGURE 3:**
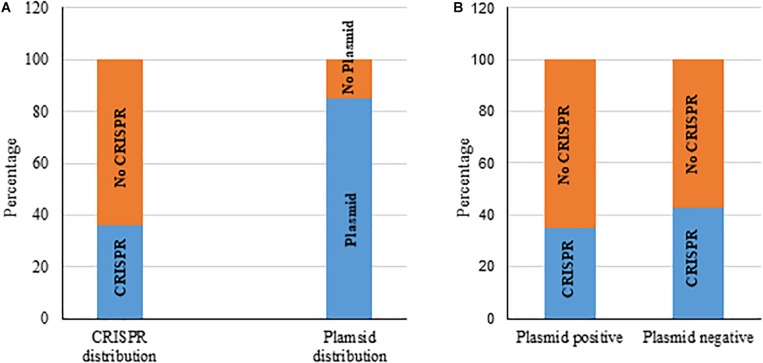
Distribution of chromosomal CRISPR-Cas system and plasmids in *K. pneumoniae* strains **(A)**. Distribution of CRISPR-Cas in plasmid-positive and -negative strains **(B)**.

We also gathered information about the presence of plasmids in those 217 *K. pneumoniae* isolates from GenBank. Most bacteria (185 of 217, 85%) carried plasmids, from 1 to 10 in number (Figure 3A and Supplementary Table S3) and 37% of these 217 had the putative CRISPR-Cas system. We found that the occurrence of chromosomal CRISPR is more in plasmid-free than plasmid-carrying strains (43 vs. 35%) (Figure 3B), a relationship that has been noted before (Li et al., 2018). We found that bacteria with chromosomal type I-E CRISPR had more plasmids (from 1 to 7 in number, most with 4–5 plasmids) whereas bacteria with chromosomal type I-E^∗^ CRISPR had less plasmids (from 0 to 4 in number, mostly only 1 or 2 plasmids or none) (Supplementary Table S3).

### Analysis of Spacers From *K. pneumoniae* Chromosomal CRISPRs

A total of 2,464 spacers were extracted from chromosomal CRISPR-positive strains. A BLASTn search with these spacers matched *K. pneumoniae* chromosomal sequences as well as different mobile elements including bacteriophages and plasmids. We identified 18 unique spacers matched sequences on plasmids (Table 3). Interestingly, 5 of these 18 spacers were from plasmid conjugative transfer region genes (*traH, traG, traT, traN, traF*) in several hundred different plasmid sequences in GenBank. Spacers matching *traH* and *traG* gene sequences from 413 and 401 different plasmids respectively were identified. One spacer matched the plasmid segregation gene *parM*, one matched the ubiquitous toxin antitoxin system gene *hok-sok* (Table 3), one matched the *SAM-methyl transferase* gene and another matched DNA sequence in a hypothetical gene located immediate upstream of *SAM-methyltransferase* on the plasmid. Three spacers matched different regions in the *DUF3560 domain-containing protein* gene, which was found in turn on ∼1,000 plasmids in GenBank. One spacer matched *ydeA*, two matched a hypothetical protein and other three in intergenic regions of plasmid sequences; one spacer matched an intergenic region with identity to 524 different plasmids (Table 3) which, in a few cases, was present in multiple times in a single plasmid sequence.

**TABLE 3:** Plasmid specific spacers in *K. pneumoniae* chromosomal CRISPR.

**Spacers**	**Target gene on plasmid^a^**	**No. of plasmid matched**	**No. of occurrence in chromosome**
TACTGCAGCAGGATGTCGTAGCCGATATAGTC	Conjugal transfer protein *traH*	413	1
ATAGAAAAGATGTGTATCGCCATCTCGGTACT	Conjugal transfer protein *traG*	401	1
TTTGGTATTTGTGCTGATTACCCGTTTCAGTA	Conjugal transfer protein *traT*	126	1
GAAATAACCGTCTTCATTTCCACCCTCCCTCA	Type-F conjugative transfer system mating-pair stabilization protein *traN*	90	1
GATACAGAATGGCTTCGTACAGCGACCGTTTG	Type-F conjugative transfer system pilin assembly protein *traF*	20	1
GTGGTTTGTTACCGTGTTGTGTGGCAAAAAGC	*hok-sok* toxin-antitoxin system	152	5
CGTGACCATTGATACGCCAATATCAGGATTAC	Plasmid segregation protein *parM*	13	1
CCGCCGTTTAATCGCGGTGATGATATCCGGCA	*SAM-dependent methyltransferase*	209	7
GTCTTCCCTGTTTGCTGCCTGCTGTCTGTCTG	*hp*, immediate upstream of *SAM-dependent methyltransferase*	405	29
GGGGACCTGCTGAACCTGCCCCCTGGTATTAA	*DUF3560* domain-containing protein	214	24
CGATAACCGGGCGTTTCGACTGAACTCACCTC	*DUF3560* domain-containing protein	351	24
TTGATACGGCGGTAACGCACATCCGGACGCTC	*DUF3560* domain-containing protein	425	1
CCGGCATCCGTCAGCTCGACGGCCAGCTGCAG	*ydeA* protein	19	24
TGATTGACGCGAAGCTGCGTTATCCCAACACC	*hp*	19	1
GCAGCATGAACGTTTCCCACTCGCCGTTCTCA	*hp*	93	1
GAGCAGGCACCCGCCGCAACGACGAAGAGCGC	Intergenic region/*hp*, (2–4 hits in same plasmid)	524	19
GAACGGAGGAATATAAGAACAAAAGCCCGCAG	Intergenic region	145	1
TCGTCTGAGTTCCGGCTTACGCCGTGCCGACA	Intergenic region	178	5

*^*a*^*hp* for hypothetical protein.*

## Discussion

In this study we have identified a putative type IV CRISPR-Cas system in the plasmids of *K. pneumoniae*. To our knowledge, this is the first comprehensive report of type IV CRISPR-Cas system in plasmids of *K. pneumoniae*, specifically in antibiotic resistance plasmids.

Type IV CRISPR-Cas was previously identified in a mega-plasmid of *Aromatoleum aromaticum* species, an aromatic-degrading β-proteobacteria found in freshwater and soils. The CRISPR-positive plasmids we describe are also very large (200–430 kb; Table 1). Like the previously reported type IV CRISPR-Cas system (Makarova et al., 2015; Enas Newire et al., 2019; Pinilla-Redondo et al., 2019) homologs of *cas* region genes *csf2*, *csf3*, *DinG helicase (csf4)*, *cas6* (*csf5*) were present, the organization of the *cas* genes was very similar and the adaptation genes *cas1* and *cas3* were absent, all typical of the previously reported type IV CRISPR-Cas. The large subunit *csf1* gene thought to be the signature gene for type IV system was absent in the CRISPR-Cas system reported here, but two additional *cas* genes (*csx3* and *cas10* homologs) and two genes of unknown functions were identified in the *cas* gene locus, any of which may compensate for the absent *csf1*. The Cas1 protein of type IV CRISPR-Cas is a Zn-finger containing protein with a weak similarity to Zn-finger sequences of Cas10 and it has been suggested that Csf1 could be a highly divergent, inactivated and N-terminally truncated Cas10-like polymerase derivative (Makarova et al., 2011a). The presence of *DinG helicase (csf4)*, only previously reported in type IV-A CRISPR-Cas (Koonin et al., 2017; Makarova et al., 2018; Pinilla-Redondo et al., 2019), further supports the designation of these plasmid systems as type IV-A CRISPR-Cas.

A preliminary study identified putative type IV CRISPR-Cas system in the IncH1B/IncFIB plasmids of Enterobacteriaceae (Enas Newire et al., 2019). In the present study most (45 of 47) of these very large CRISPR-carrying plasmids had an IncFIB replicon identified and most had an additional IncHI1B replicon. We identified only one of the IncF replicons that are thought to be typical (i.e., IncFIB_*K*_) of *K. pneumoniae* (Garcia-Fernandez et al., 2012; Villa and Carattoli, 2020). This suggests that CRISPR-positive plasmids might have originated from some other species in Enterobacteriaceae and transferred into *K. pneumoniae*. Analysis of %GC content shows that almost all CRISPR-positive plasmids have lower %GC (44–46%) than CRISPR-negative plasmids (∼50% or greater); we identified only one IncFIB_*k*_ plasmid with CRISPR (pKPM501) and this had a ‘normal’ %GC of > 51%.

Plasmids have their own genetic modules that they can utilize to exist stably in certain bacterial host by competing with other plasmids. Plasmid incompatibility is one of the such mechanism by which two plasmids with similar or related replication genes cannot co-exist in the same cell. By interfering with host replication system only one plasmid of similar type can be efficiently replicate and segregate to daughter cell and others lost form the system (Novick, 1987; Austin and Nordstrom, 1990). Acquiring antibiotic resistance genes also give plasmids the advantage to maintain over sensitive plasmids at antibiotic selection pressure (Carattoli, 2013). Plasmid mediated toxin-antitoxin (TA) module also provide another alternative to displace incompatible plasmid by toxin mediated killing of plasmid free cells (Hayes, 2003; Yamaguchi et al., 2011). For example, if a cell carries two incompatible plasmids and one plasmid encodes a TA system, then after segregation of these incompatible plasmids, only plasmid carrying TA system will be maintained into daughter cells and cells carrying the other plasmid are eliminated from the population. Similar to those systems, it was suggested that plasmid mediated type IV CRISPR-Cas system may involve in the competition between plasmids by acquiring spacers specifically targeting different plasmids (Pinilla-Redondo et al., 2019). Chromosomal CRISPR are known to acquire spacers against different MGEs (Samson et al., 2015) and many plasmid-borne CRISPR spacers we found were also directed against other plasmids, including three unique spacers targeting 100% identical sequences (the common and highly conserved *traN, traH*, and *traL* of conjugative plasmids) in more than 700 other plasmids in GenBank. Large potentially expensive plasmids such as these CRISPR-positive plasmids may need this competitive edge and may reduce the overall plasmid burden in their host bacteria. Plasmid CRISPR spacers targeting heterologous *K. pneumoniae* chromosomes may also have a role in determining the epidemiology of plasmids in this species.

Acquisition of a new plasmid produces burden to the host by reducing growth rate and lessened competitiveness of plasmid-bearing hosts under conditions that do not select for plasmid genes (San Millan and MacLean, 2017). Although this fitness-cost can be mitigated over time through compensatory evolutions, however, the initial cost associated with plasmid carriage is one of the main barrier in the acquisition, maintenance and transfer of new plasmids (San Millan and MacLean, 2017; Dionisio et al., 2019). Multiple plasmids impose more fitness-cost related to single plasmid. Acquisition of plasmid mediated CRISPR spacers targeting other plasmids and host chromosome may provide advantage in the formation of plasmid co-integrate with other plasmids or integrated into the host chromosome by homologous recombination that might facilitate the stability and compatibility of the plasmids. CRISPR-Cas defense system not only identified in plasmids but also distributed in other MGEs including bacteriophages, T7-transposable elements and integrative conjugative elements (ICEs) (Faure et al., 2019; Koonin et al., 2019). The recruitment of CRISPR-Cas defense system by different MGEs may contribute to the evolution of both MGEs and defense systems.

Several previous studies identified and analyzed chromosomal CRISPR-Cas systems in *K. pneumoniae* by analyzing 52 (Ostria-Hernandez et al., 2015), 68 (Shen et al., 2017) and 97 (Li et al., 2018) complete and draft genome sequences. Here, we analyzed 217 complete *K. pneumoniae* chromosomes available in GenBank for the distribution of CRISPR-Cas systems, their types, acquired spacers and relationship between presence and absence of CRISPR and plasmids. Consistent with previous studies, we found type I-E and type I-E^∗^ CRISPRs distributed in *K. pneumoniae* chromosomes. We found that chromosomal CRISPR-negative strains had more plasmids (Figure 3 and Supplementary Table S3) and that *K. pneumoniae* with type I-E^∗^ chromosomal CRISPR appeared to have less plasmids than those with type I-E.

Spacer sequences from chromosomal CRISPR matched different MGEs including plasmids. A total of 18 unique spacers were acquired from plasmids and many from conjugative transfer region genes, plasmid partition (*parM*) and stability genes (*hok-sok*). Acquired plasmid-specific spacers in *K. pneumoniae* chromosomal CRISPR may provide immunity against plasmids and, it has been suggested, promote or select for mobilization of important plasmid-borne antibiotic resistance genes such as *bla*_*CTX–M*_ and *bla*_*KPC*_ onto the chromosome (Huang et al., 2017). Similar phenomena have been directly observed for *Streptococcus thermophilus* CRISPR-Cas systems (Garneau et al., 2010).

Type IV CRISPR-Cas systems on plasmids lack genes for target cleavage enzymes (*cas3* or *cas10*) (Makarova et al., 2015) but we have identified a putative *cas10-like* gene in these plasmid CRISPR-Cas system in Enterobacteriaceae. They also lack key adaptation modules (*cas1* and *cas2*) but RNA processing and effector complex formation has been experimentally demonstrated for these systems in *Aromatoleum aromaticum*, in which a chromosomal type I-C CRISPR is also present (Ozcan et al., 2019). Importantly, we also noted that type IV CRISPR-Cas system-positive plasmids were found only in bacteria with chromosomal type I-E or I-E^∗^ CRISPR-Cas, suggesting cross-talk between plasmid and chromosomal CRISPR which may compensate for the lack of adaptation and target cleavage functions encoded from plasmid mediated CRISPR.

Chromosomal CRISPR-Cas systems clearly protect some bacteria from horizontally acquired mobile elements (Palmer and Gilmore, 2010; Price et al., 2016). Multi-drug resistant *Enterococcus* lacking CRISPR-Cas (Palmer and Gilmore, 2010) more readily acquire new genes and adapt to new antibiotics (Price et al., 2016). *Vibrio cholerae* that acquired phage-inducible chromosomal islands (PICI) as a defense against bacteriophages (Novick et al., 2010; Seed, 2015) now must contend with bacteriophages that have acquired CRISPR-Cas with spacers directed against chromosomal PICI to inactivate that very defense system (Naser et al., 2017). We describe here a novel type IV CRISPR-Cas that is evidently circulating in Enterobacteriaceae plasmids, predominantly within *K. pneumoniae*, and appears to have a complementary relationship with chromosomal Type I-E/I-E^∗^ CRISPRs. Plasmid CRISPR-Cas directed against other plasmids (and some *K. pneumoniae* chromosomes) provide another level of incompatibility in plasmid communities. Both plasmid and chromosomal CRISPR-Cas are evidently important determinants of the epidemiology of large antibiotic resistance plasmids in *K. pneumoniae*.

## Data Availability Statement

All datasets generated for this study are included in the article/Supplementary Material.

## Author Contributions

MK conceived and designed the study, and generated the data. MK and JI analyzed the data and wrote the manuscript.

## Conflict of Interest

The authors declare that the research was conducted in the absence of any commercial or financial relationships that could be construed as a potential conflict of interest.

## Footnotes

1 https://www.ncbi.nlm.nih.gov/genome/?term=Klebsiella+pneumoniae

2 https://crispr.i2bc.paris-saclay.fr/Server/

3 http://omics.informatics.indiana.edu/CRISPRone/

4 https://bioedit.software.informer.com/7.2/

5 https://cge.cbs.dtu.dk/services/ResFinder/

6 https://cge.cbs.dtu.dk/services/PlasmidFinder/
