# Supplementary material for: CRISPR-Cas System in Antibiotic Resistance Plasmids in Klebsiella pneumoniae
Source: Front Microbiol. 2020 Jan 10;10:2934. doi: 10.3389/fmicb.2019.02934 (PMC6965323; doi:10.3389/fmicb.2019.02934)
Supplement: Supplementary file 1 [file Data_Sheet_1.docx]

**Supplementary Information**

**for**

**CRISPR-Cas systems in antibiotic resistance plasmids in *Klebsiella pneumoniae***

**Muhammad Kamruzzaman^1*^ and Jonathan R. Iredell^1,2*^**

^1^Centre for Infectious Diseases and Microbiology, The Westmead Institute for Medical Research, The University of Sydney, Westmead, New South Wales, Australia;

^2^ Westmead Hospital, Westmead, New South Wales, Australia.

*Correspondence:

Dr. Muhammad Kamruzzaman

E-mail: [muhammad.kamruzzaman@sydney.edu.au](mailto:muhammad.kamruzzaman@sydney.edu.au)

and

Professor Jonathan R. Iredell

E-mail: [jonathan.iredell@sydney.edu.au](mailto:jonathan.iredell@sydney.edu.au)

**Supplementary Information files:**

1. **Table S1.** List of plasmids from *K. pneumoniae* strains analysed for CRISPR
2. **Table S2**. List of unique spacers found in the CRISPR-array of plasmids and their match with protospacers
3. **Table S3.** List of *K. pneumoniae* strains analysed for chromosomal CRISPR-Cas system

**Table S1. List of plasmids from *K. pneumoniae* strains analysed for CRISPR**

| Sl No. | Plasmid Name/ID | Accession No. | Plasmid Size (kb) | Plasmid GC% | CRISPR-Cas^a^ |
| --- | --- | --- | --- | --- | --- |
| 1 | plasmid2 | CP009115 | 118.061 | 52.688 | ̶̶ |
| 2 | plasmid1 | CP009116 | 94.76 | 51.2906 | ̶̶ |
| 3 | pPMK1-A | CP008930 | 187.571 | 52.4633 | ̶̶ |
| 4 | pPMK1-B | CP008931 | 111.693 | 49.1544 | ̶̶ |
| 5 | pPMK1-C | CP008932 | 69.947 | 53.9723 | ̶̶ |
| 6 | pPMK1-NDM | CP008933 | 304.526 | 47.4935 | + |
| 7 | p34618-207.543kb | CP010393 | 207.543 | 52.9004 | ̶̶ |
| 8 | p34618-71.572kb | CP010396 | 71.572 | 55.0397 | ̶̶ |
| 9 | p38544-85.403kb | CP010362 | 85.403 | 54.8154 | ̶̶ |
| 10 | p6234-198.371kb | CP010390 | 198.371 | 51.6275 | ̶̶ |
| 11 | p6234-178.193kb | CP010391 | 178.193 | 51.5284 | ̶̶ |
| 12 | unnamed_1 | CP010574 | 167.664 | 52.635 | ̶̶ |
| 13 | unnamed_2 | CP010575 | 72.864 | 54.5921 | ̶̶ |
| 14 | I | FO834904 | 95.087 | 49.9911 | ̶̶ |
| 15 | II | FO834905 | 121.703 | 49.1828 | ̶̶ |
| 16 | pCAV1392-131 | CP011577 | 130.719 | 51.6543 | ̶̶ |
| 17 | pCAV1344-78 | CP011621 | 77.808 | 53.3313 | ̶̶ |
| 18 | pKPC_CAV1344 | CP011622 | 176.497 | 52.9635 | ̶̶ |
| 19 | pCAV1344-250 | CP011623 | 250.396 | 52.6654 | ̶̶ |
| 20 | pKPC_CAV1596-78 | CP011645 | 77.801 | 45.4197 | ̶̶ |
| 21 | pKPC_CAV1596-97 | CP011646 | 96.702 | 53.8334 | ̶̶ |
| 22 | KP-plasmid1 | CP012754 | 273.628 | 46.2504 | + |
| 23 | Plasmid_A_Kpneumoniae_MS6671 | LN824134 | 279.305 | 46.7253 | + |
| 24 | Plasmid_B_Kpneumoniae_MS6671 | LN824135 | 118.323 | 52.4606 | ̶̶ |
| 25 | Plasmid_E_Kpneumoniae_MS6671 | LN824138 | 84.94 | 52.749 | ̶̶ |
| 26 | Kpneumoniae_MS6671 | LN824139 | 55.416 | 53.0569 | ̶̶ |
| 27 | pCAV1193-258 | CP013323 | 257.944 | 52.7246 | ̶̶ |
| 28 | pCAV1193-166 | CP013324 | 166.486 | 52.4398 | ̶̶ |
| 29 | pCAV1193-78 | CP013326 | 77.808 | 53.3313 | ̶̶ |
| 30 | pKp41 | CP012000 | 113.626 | 53.9234 | ̶̶ |
| 31 | pKp28 | CP011999 | 83.399 | 53.8652 | ̶̶ |
| 32 | 1 | CP013712 | 74.973 | 52.3629 | ̶̶ |
| 33 | 2 | CP013713 | 53.4 | 49.7697 | ̶̶ |
| 34 | pNDM-US | CP006661 | 140.825 | 51.9226 | ̶̶ |
| 35 | pHg | CP006662 | 85.161 | 52.7389 | ̶̶ |
| 36 | pCuAs | CP006663 | 117.755 | 51.2106 | ̶̶ |
| 37 | pKpN01-CTX | CP012988 | 190.072 | 51.7362 | ̶̶ |
| 38 | pKp02a | CP012989 | 134.064 | 51.829 | ̶̶ |
| 39 | pKpN06-CTX | CP012993 | 190.072 | 51.7378 | ̶̶ |
| 40 | unnamed 2 | CP012994 | 107.11 | 52.1315 | ̶̶ |
| 41 | unnamed 1 | CP015501 | 281.19 | 46.7997 | + |
| 42 | unnamed 2 | CP015502 | 140.874 | 52.1225 | ̶̶ |
| 43 | unnamed 3 | CP015503 | 84.941 | 52.7496 | ̶̶ |
| 44 | unnamed 4 | CP015504 | 56.23 | 52.9699 | ̶̶ |
| 45 | unnamed1 | CP015754 | 165.275 | 51.6842 | ̶̶ |
| 46 | unnamed2 | CP015755 | 109.349 | 49.3475 | ̶̶ |
| 47 | pKPN-04f | CP014756 | 121.03 | 49.277 | ̶̶ |
| 48 | pNDM-1fa | CP014757 | 54.064 | 52.2899 | ̶̶ |
| 49 | pWSZBR | CP015991 | 76.251 | 53.3265 | ̶̶ |
| 50 | pKPN-332 | CP014763 | 284.894 | 50.9003 | ̶̶ |
| 51 | pKpQIL-9b8 | CP014765 | 106.559 | 53.7261 | ̶̶ |
| 52 | pKPN-065 | CP015026 | 170.926 | 50.5839 | ̶̶ |
| 53 | pKPN-7c3 | CP015131 | 142.858 | 52.4521 | ̶̶ |
| 54 | pKPN-d90 | CP015132 | 224.457 | 52.4835 | ̶̶ |
| 55 | pKPN-fff | CP014649 | 133.484 | 52.5014 | ̶̶ |
| 56 | pKpQIL-6e6 | CP014650 | 113.639 | 53.9278 | ̶̶ |
| 57 | unnamed1 | CP016160 | 165.276 | 51.682 | ̶̶ |
| 58 | unnamed2 | CP016161 | 109.349 | 49.3466 | ̶̶ |
| 59 | pKP33 | CM004510 | 73.152 | 51.6144 | ̶̶ |
| 60 | p_IncFIB_DHQP1002001 | CP016810 | 307.743 | 51.7448 | ̶̶ |
| 61 | p_incR_DHQP1002001 | CP016812 | 65.887 | 54.9592 | ̶̶ |
| 62 | 1 | CP015823 | 205.221 | 52.8898 | ̶̶ |
| 63 | 2 | CP015824 | 103.147 | 53.6564 | ̶̶ |
| 64 | unamed | CP016815 | 212.77 | 50.1015 | ̶̶ |
| 65 | unnamed1 | CP016919 | 70.83 | 52.9183 | ̶̶ |
| 66 | unnamed2 | CP016921 | 283.369 | 46.4102 | + |
| 67 | unnamed1 | CP016922 | 103.694 | 50.9952 | ̶̶ |
| 68 | unnamed1 | CP016924 | 111.539 | 48.9255 | ̶̶ |
| 69 | unnamed2 | CP016925 | 63.291 | 52.9696 | ̶̶ |
| 70 | unnamed1 | CP016927 | 72.093 | 50.55 | ̶̶ |
| 71 | pUCLAOXA232-3.X | CP012569 | 83.73 | 53.7191 | ̶̶ |
| 72 | pUCLAOXA232-4.X | CP012570 | 111.236 | 48.9545 | ̶̶ |
| 73 | pUCLAOXA232-5.X | CP012571 | 126.863 | 52.9232 | ̶̶ |
| 74 | pUCLAOXA232-6.X | CP012572 | 163.42 | 51.7483 | ̶̶ |
| 75 | pUCLAOXA232-3 | CP012564 | 88.8 | 53.6261 | ̶̶ |
| 76 | pUCLAOXA232-4 | CP012565 | 112.059 | 48.9599 | ̶̶ |
| 77 | pUCLAOXA232-5 | CP012566 | 127.69 | 52.9204 | ̶̶ |
| 78 | pUCLAOXA232-6 | CP012567 | 196.706 | 52.4148 | ̶̶ |
| 79 | pLVPK | AY378100 | 219.385 | 50.3471 | ̶̶ |
| 80 | pK29 | EF382672 | 269.674 | 46.0916 | ̶̶ |
| 81 | pK245 | DQ449578 | 98.264 | 51.7931 | ̶̶ |
| 82 | 9 | FJ223607 | 70.655 | 54.2934 | ̶̶ |
| 83 | 12 | FJ223605 | 75.617 | 52.8029 | ̶̶ |
| 84 | pKP96 | EU195449 | 67.85 | 52.5571 | ̶̶ |
| 85 | pCTXM360 | EU938349 | 68.018 | 51.4114 | ̶̶ |
| 86 | pKF3-70 | FJ494913 | 70.057 | 52.286 | ̶̶ |
| 87 | pKF3-94 | FJ876826 | 94.219 | 51.5947 | ̶̶ |
| 88 | pKF3-140 | FJ876827 | 147.416 | 52.4638 | ̶̶ |
| 89 | pKpQIL | GU595196 | 113.637 | 53.9261 | ̶̶ |
| 90 | pKP048 | FJ628167 | 151.188 | 51.3295 | ̶̶ |
| 91 | pNL194 | GU585907 | 79.307 | 53.1202 | ̶̶ |
| 92 | pc15-k | HQ202266 | 95.626 | 52.0852 | ̶̶ |
| 93 | pUUH239.2 | CP002474 | 220.824 | 52.8185 | ̶̶ |
| 94 | pR55 | JQ010984 | 170.81 | 52.9998 | ̶̶ |
| 95 | pNDM-MAR | JN420336 | 267 | 46.01 | ̶̶ |
| 96 | pNDM-KN | JN157804 | 162.746 | 51.8366 | ̶̶ |
| 97 | pOXA-48 | JN626286 | 61.881 | 51.1062 | ̶̶ |
| 98 | pKpQIL-IT | JN233705 | 115.3 | 53.8508 | ̶̶ |
| 99 | pNDM10469 | JN861072 | 137.813 | 51.9704 | ̶̶ |
| 100 | pNDM-HN380 | JX104760 | 54.035 | 49.0386 | ̶̶ |
| 101 | pKPN101-IT | JX283456 | 107.748 | 52.7063 | ̶̶ |
| 102 | pKpS90 | JX461340 | 53.286 | 49.5909 | ̶̶ |
| 103 | pKDO1 | JX424423 | 127.508 | 52.7959 | ̶̶ |
| 104 | pKPN_CZ | JX424424 | 207.819 | 53.3849 | ̶̶ |
| 105 | 1 | CP017386 | 155.781 | 53.0244 | ̶̶ |
| 106 | 2 | CP017387 | 225.962 | 51.0267 | ̶̶ |
| 107 | pSg1-NDM | CP011839 | 90.103 | 53.5343 | ̶̶ |
| 108 | pSg1-1 | CP012427 | 126.466 | 49.248 | ̶̶ |
| 109 | pSg1-2 | CP012428 | 54.744 | 48.8583 | ̶̶ |
| 110 | pCAV1016-90 | CP017935 | 89.621 | 54.8108 | ̶̶ |
| 111 | pCAV1016-76 | CP017936 | 76.186 | 52.6868 | ̶̶ |
| 112 | pMNCRE78_4 | CP018430 | 208.225 | 52.8789 | ̶̶ |
| 113 | pMNCRE78_3 | CP018432 | 52.059 | 48.1415 | ̶̶ |
| 114 | pMNCRE69_4 | CP018424 | 208.225 | 52.8789 | ̶̶ |
| 115 | pMNCRE69_3 | CP018426 | 52.058 | 48.1425 | ̶̶ |
| 116 | pMNCRE53_4 | CP018434 | 208.225 | 52.8793 | ̶̶ |
| 117 | pMNCRE53_3 | CP018436 | 52.058 | 48.1386 | ̶̶ |
| 118 | pCAV1453-208 | CP018355 | 207.543 | 52.9004 | ̶̶ |
| 119 | pKp_Goe_414-2 | CP018338 | 202.175 | 49.5229 | ̶̶ |
| 120 | pKp_Goe_414-1 | CP018339 | 204.862 | 44.8194 | + |
| 121 | pKp_Goe_414-3 | CP018340 | 81.939 | 52.4036 | ̶̶ |
| 122 | pKp_Goe_414-4 | CP018341 | 81.641 | 53.9018 | ̶̶ |
| 123 | pKp_Goe_414-5 | CP018342 | 63.588 | 51.2235 | ̶̶ |
| 124 | pKp_Goe_414-6 | CP018343 | 57.266 | 52.1199 | ̶̶ |
| 125 | pKp_Goe_629-1 | CP018365 | 260.772 | 52.6736 | ̶̶ |
| 126 | pKp_Goe_629-2 | CP018366 | 94.434 | 55.6876 | ̶̶ |
| 127 | pKp_Goe_795-1 | CP018460 | 232.181 | 50.6282 | ̶̶ |
| 128 | pKp_Goe_795-2 | CP018461 | 63.593 | 51.2242 | ̶̶ |
| 129 | pKp_Goe_917-1 | CP018441 | 180.027 | 52.0777 | ̶̶ |
| 130 | pKp_Goe_917-2 | CP018443 | 50.611 | 51.5145 | ̶̶ |
| 131 | pKp_Goe_208-1 | CP018448 | 90.685 | 53.1709 | ̶̶ |
| 132 | pKp_Goe_208-2 | CP018449 | 67.101 | 50.5775 | ̶̶ |
| 133 | unnamed | CP018455 | 162.552 | 53.6345 | ̶̶ |
| 134 | pKp_Goe_070-1 | CP018451 | 90.684 | 53.1692 | ̶̶ |
| 135 | pKp_Goe_070-2 | CP018452 | 67.1 | 50.5753 | ̶̶ |
| 136 | pCAV1417-67 | CP018350 | 66.92 | 51.9785 | ̶̶ |
| 137 | pCAV1417-185 | CP018351 | 184.94 | 52.4549 | ̶̶ |
| 138 | pKPC_CAV1042-89 | CP018669 | 88.688 | 53.9239 | ̶̶ |
| 139 | pCAV1042-183 | CP018670 | 183.432 | 52.0062 | ̶̶ |
| 140 | pKp_Goe_473-1 | CP018687 | 246.757 | 45.1185 | + |
| 141 | pKp_Goe_473-5 | CP018688 | 75.838 | 52.5475 | ̶̶ |
| 142 | pKp_Goe_473-2 | CP018689 | 96.07 | 53.1394 | ̶̶ |
| 143 | pKp_Goe_473-3 | CP018690 | 63.589 | 51.2243 | ̶̶ |
| 144 | pKp_Goe_473-4 | CP018691 | 61.007 | 51.3564 | ̶̶ |
| 145 | pKp_Goe_024-1 | CP018702 | 246.753 | 45.1176 | + |
| 146 | pKp_Goe_024-5 | CP018703 | 66.149 | 52.7264 | ̶̶ |
| 147 | pKp_Goe_024-2 | CP018704 | 96.073 | 53.1398 | ̶̶ |
| 148 | pKp_Goe_024-4 | CP018705 | 61.01 | 51.3555 | ̶̶ |
| 149 | pKp_Goe_024-3 | CP018706 | 63.588 | 51.2251 | ̶̶ |
| 150 | pKp_Goe_026-1 | CP018708 | 246.756 | 45.1174 | + |
| 151 | pKp_Goe_026-5 | CP018709 | 73.019 | 52.5493 | ̶̶ |
| 152 | pKp_Goe_026-2 | CP018710 | 96.073 | 53.1398 | ̶̶ |
| 153 | pKp_Goe_026-4 | CP018711 | 61.009 | 51.3564 | ̶̶ |
| 154 | pKp_Goe_026-3 | CP018712 | 63.583 | 51.2212 | ̶̶ |
| 155 | pKp_Goe_021-1 | CP018714 | 246.756 | 45.1174 | + |
| 156 | pKp_Goe_021-5 | CP018715 | 80.892 | 52.8198 | ̶̶ |
| 157 | pKp_Goe_021-2 | CP018716 | 96.074 | 53.1392 | ̶̶ |
| 158 | pKp_Goe_021-3 | CP018717 | 63.589 | 51.2243 | ̶̶ |
| 159 | pKp_Goe_021-4 | CP018718 | 61.01 | 51.3555 | ̶̶ |
| 160 | pKp_Goe_304-1 | CP018720 | 246.757 | 45.1177 | + |
| 161 | pKp_Goe_304-5 | CP018721 | 73.597 | 52.2793 | ̶̶ |
| 162 | pKp_Goe_304-2 | CP018722 | 96.073 | 53.1398 | ̶̶ |
| 163 | pKp_Goe_304-3 | CP018723 | 63.588 | 51.2251 | ̶̶ |
| 164 | pKp_Goe_304-4 | CP018724 | 61.012 | 51.3571 | ̶̶ |
| 165 | pCAV1217-71 | CP018674 | 70.606 | 52.4148 | ̶̶ |
| 166 | pKPC_CAV1217 | CP018675 | 181.436 | 52.9338 | ̶̶ |
| 167 | pKp_Goe_832-1 | CP018696 | 246.755 | 45.1176 | + |
| 168 | pKp_Goe_832-5 | CP018697 | 79.806 | 52.5875 | ̶̶ |
| 169 | pKp_Goe_832-2 | CP018698 | 96.072 | 53.1393 | ̶̶ |
| 170 | pKp_Goe_832-4 | CP018699 | 61.01 | 51.3555 | ̶̶ |
| 171 | pKp_Goe_832-3 | CP018700 | 63.588 | 51.2282 | ̶̶ |
| 172 | pKp_Goe_588-1 | CP018693 | 180.027 | 52.0777 | ̶̶ |
| 173 | pKp_Goe_588-2 | CP018694 | 50.609 | 51.5146 | ̶̶ |
| 174 | pKp_Goe_641-2 | CP018736 | 63.589 | 51.2227 | ̶̶ |
| 175 | pKp_Goe_641-1 | CP018737 | 72.952 | 52.7511 | ̶̶ |
| 176 | unitig_1 | CP018817 | 140.825 | 51.9226 | ̶̶ |
| 177 | unitig_2 | CP018818 | 117.755 | 51.2055 | ̶̶ |
| 178 | unitig_3 | CP018819 | 85.161 | 52.7354 | ̶̶ |
| 179 | p35657-1 | CP015135 | 158.741 | 51.2426 | ̶̶ |
| 180 | pKp1756 | CP019220 | 73.952 | 52.7572 | ̶̶ |
| 181 | CN1_p1 | CP015383 | 182.846 | 51.5888 | ̶̶ |
| 182 | NY9_p1 | CP015386 | 199.497 | 52.9336 | ̶̶ |
| 183 | NY9_p2 | CP015387 | 139.933 | 54.0423 | ̶̶ |
| 184 | NY9_p3 | CP015388 | 89.067 | 52.5458 | ̶̶ |
| 185 | NY9_p4 | CP015389 | 53.3 | 51.3996 | ̶̶ |
| 186 | CR14_p1 | CP015393 | 202.696 | 52.5338 | ̶̶ |
| 187 | CR14_p2 | CP015394 | 154.343 | 51.7192 | ̶̶ |
| 188 | CR14_p3 | CP015395 | 116.419 | 53.7601 | ̶̶ |
| 189 | CR14_p4 | CP015396 | 110.092 | 49.6785 | ̶̶ |
| 190 | unnamed1 | CP017986 | 244.706 | 45.1166 | + |
| 191 | unnamed2 | CP017987 | 117.168 | 53.2159 | ̶̶ |
| 192 | unnamed3 | CP017988 | 73.221 | 52.5764 | ̶̶ |
| 193 | unnamed4 | CP017989 | 61.011 | 51.3547 | ̶̶ |
| 194 | pKp_Goe_579-1 | CP018313 | 245.975 | 45.0899 | + |
| 195 | pKp_Goe_579-6 | CP018314 | 68.601 | 52.2645 | ̶̶ |
| 196 | pKp_Goe_579-3 | CP018315 | 63.587 | 51.2227 | ̶̶ |
| 197 | pKp_Goe_579-4 | CP018316 | 61.012 | 51.3587 | ̶̶ |
| 198 | pKp_Goe_579-2 | CP018318 | 96.082 | 53.1452 | ̶̶ |
| 199 | unnamed1 | CP014295 | 137.267 | 51.6665 | ̶̶ |
| 200 | unnamed11 | CP014296 | 76.205 | 54.8639 | ̶̶ |
| 201 | unnamed13 | CP014297 | 90.274 | 54.6968 | ̶̶ |
| 202 | unnamed2 | CP014298 | 70.438 | 51.5475 | ̶̶ |
| 203 | KPN207_p2 | LT216438 | 117.916 | 53.5084 | ̶̶ |
| 204 | KPN207_p3 | LT216439 | 214.718 | 52.5466 | ̶̶ |
| 205 | unitig_1 | CP020062 | 152.697 | 50.4732 | ̶̶ |
| 206 | unitig_2 | CP020063 | 109.019 | 49.1575 | ̶̶ |
| 207 | unitig_3 | CP020064 | 83.376 | 54.0323 | ̶̶ |
| 208 | unitig_4 | CP020065 | 74.54 | 51.5025 | ̶̶ |
| 209 | unitig_5 | CP020066 | 72.663 | 53.422 | ̶̶ |
| 210 | unitig_1 | CP020068 | 276.46 | 46.5977 | + |
| 211 | unitig_2 | CP020069 | 213.013 | 52.363 | ̶̶ |
| 212 | tig00000002 | CP020072 | 209.424 | 52.8831 | ̶̶ |
| 213 | tig00000001 | CP020109 | 169.952 | 52.6966 | ̶̶ |
| 214 | tig00000002 | CP020110 | 73.467 | 54.7293 | ̶̶ |
| 215 | pK66-45-1 | CP020902 | 338.512 | 47.8382 | + |
| 216 | pK66-45-2 | CP020903 | 200.365 | 52.3924 | ̶̶ |
| 217 | pK66-45-3 | CP020904 | 120.533 | 52.2911 | ̶̶ |
| 218 | pBK13043-1 | CP020838 | 232.54 | 53.6248 | ̶̶ |
| 219 | pBK13043-2 | CP020839 | 57.58 | 46.8062 | ̶̶ |
| 220 | pKPN1482-1 | CP020842 | 180.21 | 52.3323 | ̶̶ |
| 221 | pKPN1482-2 | CP020843 | 97.202 | 51.9567 | ̶̶ |
| 222 | pKPN1482-3 | CP020844 | 74.177 | 51.1857 | ̶̶ |
| 223 | pKPN528-1 | CP020854 | 292.471 | 46.3454 | + |
| 224 | pKPN528-2 | CP020855 | 221.428 | 52.5349 | ̶̶ |
| 225 | pKPN528-3 | CP020856 | 76.158 | 53.3601 | ̶̶ |
| 226 | pKp145/11b | KX118608 | 68.582 | 50.9667 | ̶̶ |
| 227 | pKp145/11a | KX154765 | 50.8 | 52.4291 | ̶̶ |
| 228 | pKp196; TIET-4200 | KX397572 | 55.902 | 52.7709 | ̶̶ |
| 229 | pKP148,PINH-4900 | KX062091 | 52.536 | 52.4688 | ̶̶ |
| 230 | pKp314/11a | KX276209 | 54.609 | 52.896 | ̶̶ |
| 231 | tig00000001 | CP021540 | 201.874 | 53.0152 | ̶̶ |
| 232 | tig00000002 | CP021541 | 111.531 | 53.0131 | ̶̶ |
| 233 | tig00000000 | CP021544 | 208.223 | 52.8794 | ̶̶ |
| 234 | tig00000001 | CP021545 | 125.536 | 53.6165 | ̶̶ |
| 235 | tig00000002 | CP021546 | 74.66 | 45.2049 | ̶̶ |
| 236 | tig00001160 | CP021686 | 183.376 | 51.1043 | ̶̶ |
| 237 | tig00001186 | CP021687 | 96.814 | 53.233 | ̶̶ |
| 238 | tig00001189 | CP021688 | 90.199 | 51.8964 | ̶̶ |
| 239 | tig00000161 | CP021697 | 191.266 | 49.7809 | ̶̶ |
| 240 | tig00000183 | CP021698 | 87.411 | 51.9157 | ̶̶ |
| 241 | tig00000727 | CP021699 | 354.705 | 47.8519 | ̶̶ |
| 242 | tig00000000 | CP021713 | 208.224 | 52.8796 | ̶̶ |
| 243 | tig00000001 | CP021714 | 106.541 | 49.4955 | ̶̶ |
| 244 | tig00000001_p1 | CP021709 | 186.758 | 50.8369 | ̶̶ |
| 245 | tig00000853 | CP021710 | 214.114 | 52.3422 | ̶̶ |
| 246 | tig00000856 | CP021711 | 78.638 | 51.765 | ̶̶ |
| 247 | tig00000857 | CP021712 | 70.457 | 52.9018 | ̶̶ |
| 248 | tig00000001 | CP021741 | 89.621 | 54.8108 | ̶̶ |
| 249 | tig00000002jNODE_23 | CP021742 | 76.179 | 52.6904 | ̶̶ |
| 250 | unitig_1 | CP021752 | 209.55 | 52.8471 | ̶̶ |
| 251 | unitig_2 | CP021753 | 116.187 | 53.308 | ̶̶ |
| 252 | unitig_3 | CP021754 | 74.751 | 45.2061 | ̶̶ |
| 253 | tig00000001 | CP021758 | 121.057 | 53.2427 | ̶̶ |
| 254 | tig00000003 | CP021760 | 54.404 | 53.1762 | ̶̶ |
| 255 | tig00000004 | CP021761 | 79.767 | 52.4766 | ̶̶ |
| 256 | tig00000500_pilon | CP021834 | 212.837 | 52.777 | ̶̶ |
| 257 | tig00000516_pilon | CP021835 | 210.045 | 53.2524 | ̶̶ |
| 258 | tig00000001_pilon | CP021856 | 146.168 | 53.0184 | ̶̶ |
| 259 | tig00000002_pilon | CP021857 | 106.331 | 54.1216 | ̶̶ |
| 260 | tig00000009_pilon | CP021861 | 79.489 | 45.6201 | ̶̶ |
| 261 | tig00000002_u | CP021958 | 128.92 | 51.1201 | ̶̶ |
| 262 | tig00000003 | CP021959 | 89.382 | 52.816 | ̶̶ |
| 263 | tig00000006 | CP021961 | 97.389 | 52.8797 | ̶̶ |
| 264 | tig00000008 | CP021962 | 132.217 | 49.1155 | ̶̶ |
| 265 | tig00000209 | CP021940 | 112.868 | 48.959 | ̶̶ |
| 266 | tig00000217 | CP021941 | 54.064 | 52.2917 | ̶̶ |
| 267 | tig00000218j2847_linear | CP021942 | 76.739 | 52.2785 | ̶̶ |
| 268 | tig00000194 | CP021945 | 112.868 | 48.959 | ̶̶ |
| 269 | tig00000195 | CP021946 | 120.345 | 53.5826 | ̶̶ |
| 270 | tig00000200 | CP021947 | 54.064 | 52.2917 | ̶̶ |
| 271 | tig00000216_u | CP021949 | 81.698 | 52.7173 | ̶̶ |
| 272 | tig00000168_pilon | CP021951 | 181.589 | 51.0835 | ̶̶ |
| 273 | tig00000169_pilon | CP021952 | 176.349 | 51.9408 | ̶̶ |
| 274 | tig00000185_pilon | CP021953 | 69.541 | 52.963 | ̶̶ |
| 275 | unitig_1_pilon | CP021956 | 153.527 | 51.7069 | ̶̶ |
| 276 | unitig_3j2_linear_pilon | CP021957 | 72.122 | 52.9824 | ̶̶ |
| 277 | p1605752FIB | CP022125 | 187.721 | 51.0774 | ̶̶ |
| 278 | p1605752AC2 | CP022126 | 140.133 | 51.9656 | ̶̶ |
| 279 | p1605752FIB_2 | CP022128 | 111.692 | 49.153 | ̶̶ |
| 280 | p704SK6_1 | CP022144 | 209.651 | 45.1746 | ̶̶ |
| 281 | p704SK6_2 | CP022145 | 197.67 | 52.8583 | ̶̶ |
| 282 | p704SK6_3 | CP022146 | 65.998 | 50.9667 | ̶̶ |
| 283 | p704SK6_4 | CP022147 | 63.605 | 51.2098 | ̶̶ |
| 284 | pBIC-1a | CP022574 | 170.415 | 52.7272 | ̶̶ |
| 285 | unnamed2 | CP023942 | 187.926 | 53.437 | ̶̶ |
| 286 | unnamed1 | CP023943 | 196.733 | 52.1727 | ̶̶ |
| 287 | unnamed2 | CP023947 | 153.385 | 51.7593 | ̶̶ |
| 288 | unnamed1 | CP023948 | 219.35 | 52.062 | ̶̶ |
| 289 | unnamed3 | CP023914 | 106.853 | 55.4322 | ̶̶ |
| 290 | unnamed2 | CP023916 | 130.348 | 51.0572 | ̶̶ |
| 291 | unnamed1 | CP023917 | 164.607 | 49.6923 | ̶̶ |
| 292 | unnamed | CP023918 | 106.914 | 50.955 | ̶̶ |
| 293 | unnamed2 | CP023922 | 129.106 | 52.1804 | ̶̶ |
| 294 | unnamed1 | CP023923 | 140.557 | 50.7332 | ̶̶ |
| 295 | unnamed2 | CP023910 | 100.207 | 52.2349 | ̶̶ |
| 296 | unnamed1 | CP023912 | 167.814 | 52.7268 | ̶̶ |
| 297 | unnamed3 | CP023950 | 99.251 | 49.5753 | ̶̶ |
| 298 | unnamed1 | CP023952 | 207.802 | 53.0014 | ̶̶ |
| 299 | unnamed2 | CP023953 | 197.671 | 53.4302 | ̶̶ |
| 300 | unnamed3 | CP023927 | 88.025 | 51.2934 | ̶̶ |
| 301 | unnamed1 | CP023928 | 113.639 | 53.9295 | ̶̶ |
| 302 | unnamed2 | CP023929 | 112.26 | 49.4272 | ̶̶ |
| 303 | unnamed3 | CP023934 | 77.986 | 53.2352 | ̶̶ |
| 304 | unnamed1 | CP023937 | 279.104 | 52.2812 | ̶̶ |
| 305 | unnamed2 | CP023938 | 91.069 | 55.3416 | ̶̶ |
| 306 | unnamed1 | CP024192 | 221.606 | 53.3934 | ̶̶ |
| 307 | unnamed2 | CP024193 | 147.945 | 52.2802 | ̶̶ |
| 308 | unnamed3 | CP024194 | 89.345 | 50.6811 | ̶̶ |
| 309 | unnamed1 | CP024490 | 71.104 | 52.0927 | ̶̶ |
| 310 | unnamed1 | CP024483 | 243.634 | 52.4935 | ̶̶ |
| 311 | unnamed2 | CP024484 | 71.104 | 52.0927 | ̶̶ |
| 312 | unnamed1 | CP024497 | 212.097 | 44.192 | ̶̶ |
| 313 | pMR0617aac | CP024459 | 211.313 | 50.7976 | ̶̶ |
| 314 | pMRSN480738_112.7 | CP024460 | 112.745 | 49.2332 | ̶̶ |
| 315 | pMR0617tem | CP024461 | 77.269 | 53.0161 | ̶̶ |
| 316 | unnamed1 | CP024564 | 187.611 | 53.0662 | ̶̶ |
| 317 | unnamed2 | CP024565 | 147.932 | 52.2808 | ̶̶ |
| 318 | unnamed3 | CP024566 | 89.345 | 50.6811 | ̶̶ |
| 319 | unnamed1 | CP024543 | 110.374 | 52.2306 | ̶̶ |
| 320 | unnamed2 | CP024544 | 71.587 | 54.197 | ̶̶ |
| 321 | unnamed1 | CP024507 | 310.025 | 47.3933 | + |
| 322 | unnamed2 | CP024508 | 228.353 | 52.1333 | ̶̶ |
| 323 | unnamed3 | CP024509 | 140.704 | 52.5728 | ̶̶ |
| 324 | unnamed4 | CP024510 | 98.344 | 52.8095 | ̶̶ |
| 325 | unnamed1 | CP024516 | 227.807 | 52.264 | ̶̶ |
| 326 | unnamed2 | CP024517 | 212.079 | 44.1901 | ̶̶ |
| 327 | unnamed1 | CP024522 | 221.606 | 53.393 | ̶̶ |
| 328 | unnamed2 | CP024523 | 147.932 | 52.2808 | ̶̶ |
| 329 | unnamed3 | CP024524 | 89.345 | 50.6799 | ̶̶ |
| 330 | unnamed1 | CP024529 | 221.606 | 53.3943 | ̶̶ |
| 331 | unnamed2 | CP024530 | 147.932 | 52.2808 | ̶̶ |
| 332 | unnamed3 | CP024531 | 89.345 | 50.6811 | ̶̶ |
| 333 | unnamed1 | CP024536 | 187.611 | 53.0651 | ̶̶ |
| 334 | unnamed2 | CP024537 | 147.932 | 52.2821 | ̶̶ |
| 335 | unnamed3 | CP024538 | 89.345 | 50.6811 | ̶̶ |
| 336 | unnamed1 | CP024546 | 110.374 | 52.2306 | ̶̶ |
| 337 | unnamed2 | CP024547 | 71.587 | 54.197 | ̶̶ |
| 338 | unnamed1 | CP024550 | 187.611 | 53.0651 | ̶̶ |
| 339 | unnamed2 | CP024551 | 147.932 | 52.2808 | ̶̶ |
| 340 | unnamed3 | CP024552 | 89.345 | 50.6811 | ̶̶ |
| 341 | unnamed1 | CP024557 | 217.685 | 53.3904 | ̶̶ |
| 342 | unnamed2 | CP024558 | 147.945 | 52.2802 | ̶̶ |
| 343 | unnamed3 | CP024559 | 89.345 | 50.6811 | ̶̶ |
| 344 | unnamed1 | CP024571 | 187.611 | 53.0662 | ̶̶ |
| 345 | unnamed2 | CP024572 | 147.932 | 52.2808 | ̶̶ |
| 346 | unnamed3 | CP024573 | 90.173 | 50.6948 | ̶̶ |
| 347 | unnamed1 | CP024500 | 243.62 | 52.4887 | ̶̶ |
| 348 | unnamed2 | CP024501 | 212.195 | 44.2004 | ̶̶ |
| 349 | pCRKP-1215_1 | CP024839 | 130.922 | 52.6268 | ̶̶ |
| 350 | pCRKP-1215_2 | CP024840 | 96.185 | 52.6756 | ̶̶ |
| 351 | pCRKP-1215_3 | CP024841 | 72.689 | 52.0491 | ̶̶ |
| 352 | pCRKP-2297_1 | CP024835 | 112.15 | 52.2782 | ̶̶ |
| 353 | pCRKP-2297_2 | CP024836 | 96.185 | 52.6756 | ̶̶ |
| 354 | pCRKP-2297_3 | CP024837 | 69.628 | 52.173 | ̶̶ |
| 355 | pAUSMDU3562-1 | CP025006 | 167.373 | 52.9882 | ̶̶ |
| 356 | pAUSMDU3562-2 | CP025007 | 51.423 | 47.9144 | ̶̶ |
| 357 | pAUSMDU8119-1 | CP025009 | 176.049 | 52.9137 | ̶̶ |
| 358 | pAUSMDU8119-2 | CP025010 | 118.202 | 53.9103 | ̶̶ |
| 359 | pNU-CRE047_1 | CP025038 | 199.686 | 53.1304 | ̶̶ |
| 360 | pNU-CRE047_2 | CP025039 | 146.689 | 54.0906 | ̶̶ |
| 361 | pNU-CRE047_3 | CP025040 | 83.541 | 53.9699 | ̶̶ |
| 362 | pNU-CRE047_4 | CP025041 | 63.772 | 47.0426 | ̶̶ |
| 363 | pSGH10 | CP025081 | 231.583 | 50.1535 | ̶̶ |
| 364 | NR5632_p1 | CP025144 | 204.123 | 52.9833 | ̶̶ |
| 365 | NR5632_p2 | CP025145 | 149.158 | 52.9425 | ̶̶ |
| 366 | KP1768_p1 | CP025141 | 204.734 | 52.9819 | ̶̶ |
| 367 | KP1768_p2 | CP025142 | 152.23 | 52.8746 | ̶̶ |
| 368 | KP1766_p1 | CP025147 | 205.953 | 52.9708 | ̶̶ |
| 369 | KP1766_p2 | CP025148 | 161.986 | 53.4077 | ̶̶ |
| 370 | p18ES-342 | CM008881 | 332.674 | 46.9739 | + |
| 371 | pNDM_18ES | CM008882 | 110.432 | 54.8926 | ̶̶ |
| 372 | p69-1 | CP025457 | 223.274 | 51.8242 | ̶̶ |
| 373 | p69-2 | CP025458 | 128.563 | 54.5064 | ̶̶ |
| 374 | p187-1 | CP025467 | 246.557 | 47.415 | ̶̶ |
| 375 | p187-2 | CP025468 | 129.684 | 52.6187 | ̶̶ |
| 376 | p187-4 | CP025470 | 106.402 | 52.4567 | ̶̶ |
| 377 | p44-1 | CP025462 | 261.706 | 47.5022 | + |
| 378 | p44-2 | CP025463 | 161.58 | 53.9473 | ̶̶ |
| 379 | p002SK2_A | CP025516 | 159.714 | 52.5208 | ̶̶ |
| 380 | p002SK2_B | CP025517 | 77.809 | 53.0286 | ̶̶ |
| 381 | pDT1 | CP019078 | 70.987 | 50.5332 | ̶̶ |
| 382 | pDT12 | CP019080 | 70.987 | 50.5304 | ̶̶ |
| 383 | pKP8-1 | CP025637 | 86.207 | 53.1082 | ̶̶ |
| 384 | pKP8-2 | CP025638 | 153.586 | 48.5956 | ̶̶ |
| 385 | pKP9 | CP025640 | 190.47 | 50.3407 | ̶̶ |
| 386 | pKP | CP025632 | 169.967 | 51.0393 | ̶̶ |
| 387 | pKP6-1 | CP025634 | 172.768 | 49.8345 | ̶̶ |
| 388 | pKP6-2 | CP025635 | 110.182 | 49.0842 | ̶̶ |
| 389 | pKP7 | CP025642 | 197.806 | 50.3306 | ̶̶ |
| 390 | pKp81_1 | CP025817 | 101.557 | 52.3726 | ̶̶ |
| 391 | pKPN-bbef | CP026172 | 243.967 | 46.2936 | + |
| 392 | pKPN-c4ac | CP026173 | 64.271 | 53.6494 | ̶̶ |
| 393 | pKPN-0d7f | CP026174 | 93.68 | 54.2784 | ̶̶ |
| 394 | pKPC-0cc9 | CP026175 | 172.259 | 51.945 | ̶̶ |
| 395 | pKPC-224e | CP026179 | 237.571 | 51.4112 | ̶̶ |
| 396 | pKPN-9729 | CP026180 | 97.198 | 51.9692 | ̶̶ |
| 397 | pKPN-6a23 | CP026181 | 94.405 | 53.6084 | ̶̶ |
| 398 | pKPN-0c4e | CP026182 | 61.178 | 52.6774 | ̶̶ |
| 399 | pKPN-af73 | CP026184 | 60.046 | 52.0451 | ̶̶ |
| 400 | pKPN-3967 | CP026186 | 373.179 | 48.0322 | + |
| 401 | pKPC-e937 | CP026394 | 63.725 | 53.5708 | ̶̶ |
| 402 | pKPC-edb7 | CP026395 | 80.642 | 51.7981 | ̶̶ |
| 403 | pKPN-8c6e | CP026396 | 144.072 | 51.6061 | ̶̶ |
| 404 | pKPN-10f7 | CP026397 | 220.406 | 52.3538 | ̶̶ |
| 405 | pKPN-edaa | CP026398 | 249.238 | 46.5206 | + |
| 406 | p1 | CP026587 | 215.697 | 50.4536 | ̶̶ |
| 407 | p2 | CP026588 | 126.149 | 52.2517 | ̶̶ |
| 408 | p3 | CP026589 | 89.247 | 53.2567 | ̶̶ |
| 409 | tig00000080_pilon | CP026752 | 211.813 | 52.3561 | ̶̶ |
| 410 | tig00000084_pilon | CP026753 | 70.73 | 52.8305 | ̶̶ |
| 411 | unnamed1 | CP014121 | 200.203 | 52.5277 | ̶̶ |
| 412 | pGN-2 | CP019161 | 261.986 | 49.995 | ̶̶ |
| 413 | unnamed1 | CP025212 | 145.759 | 52.2499 | ̶̶ |
| 414 | unnamed2 | CP025213 | 103.957 | 52.4534 | ̶̶ |
| 415 | unnamed3 | CP025214 | 87.395 | 52.1357 | ̶̶ |
| 416 | unnamed4 | CP025215 | 68.637 | 45.694 | ̶̶ |
| 417 | unnamed3 | CP027150 | 65.684 | 55.3011 | ̶̶ |
| 418 | unnamed1 | CP027152 | 208.849 | 52.9028 | ̶̶ |
| 419 | unnamed4 | CP027156 | 184.525 | 44.5983 | + |
| 420 | unnamed2 | CP027158 | 113.639 | 53.9295 | ̶̶ |
| 421 | unnamed1 | CP027161 | 207.543 | 52.8994 | ̶̶ |
| 422 | p48896_1 | CP024430 | 131.243 | 51.7948 | ̶̶ |
| 423 | p48896_2 | CP024431 | 114.815 | 53.1734 | ̶̶ |
| 424 | p48896_4 | CP024433 | 55.118 | 52.8357 | ̶̶ |
| 425 | unnamed | CP027603 | 457.578 | 49.6009 | ̶̶ |
| 426 | pKPC2_095084 | CP027067 | 120.074 | 53.8751 | ̶̶ |
| 427 | pGMI16-005_01 | CP028181 | 227.967 | 53.4235 | ̶̶ |
| 428 | pGMI16-006_1 | CP028177 | 116.768 | 52.5272 | ̶̶ |
| 429 | pGMI16-006_2 | CP028178 | 100.222 | 52.245 | ̶̶ |
| 430 | pNH25.1 | CP024875 | 227.692 | 51.3817 | ̶̶ |
| 431 | pNH25.2 | CP024876 | 129.299 | 53.4567 | ̶̶ |
| 432 | pNH25.3 | CP024877 | 109.72 | 48.858 | ̶̶ |
| 433 | pNH25.4 | CP024878 | 73.339 | 52.2969 | ̶̶ |
| 434 | pKPNH54.1 | CP024917 | 126.018 | 52.7004 | ̶̶ |
| 435 | pKPNH54.2 | CP024918 | 81.261 | 49.4038 | ̶̶ |
| 436 | pSCM96-1 | CP028717 | 134.869 | 52.6363 | ̶̶ |
| 437 | unnamed1 | CP028929 | 283.371 | 46.4105 | + |
| 438 | unnamed2 | CP028930 | 103.694 | 50.9962 | ̶̶ |
| 439 | unnamed3 | CP028931 | 70.829 | 52.919 | ̶̶ |
| 440 | pCMY2_085072 | CP028804 | 323.934 | 52.6082 | ̶̶ |
| 441 | pKPC2_085072 | CP028805 | 131.028 | 51.8019 | ̶̶ |
| 442 | pKPC2_020036 | CP028582 | 149.258 | 53.5475 | ̶̶ |
| 443 | p1_040035 | CP028794 | 54.847 | 52.8069 | ̶̶ |
| 444 | pKPC2_040035 | CP028796 | 112.467 | 54.5093 | ̶̶ |
| 445 | pKPC2_020030 | CP028790 | 104.81 | 53.0541 | ̶̶ |
| 446 | pOXA1_020030 | CP028791 | 288.222 | 46.8521 | + |
| 447 | pQnrS1_020030 | CP028792 | 125.009 | 51.9955 | ̶̶ |
| 448 | unnamed1 | CP028954 | 214.704 | 51.6055 | ̶̶ |
| 449 | unnamed2 | CP028955 | 134.346 | 52.8933 | ̶̶ |
| 450 | pMR0617ndm | CP024039 | 125.285 | 50.2694 | ̶̶ |
| 451 | pMR0617ctx | CP024040 | 122.082 | 52.2944 | ̶̶ |
| 452 | unnamed1 | CP028991 | 85.698 | 51.4971 | ̶̶ |
| 453 | unnamed2 | CP028992 | 68.953 | 52.4705 | ̶̶ |
| 454 | unnamed6 | CP028995 | 152.176 | 51.4641 | ̶̶ |
| 455 | unnamed5 | CP028996 | 176.325 | 51.9059 | ̶̶ |
| 456 | unnamed4 | CP028997 | 109.938 | 49.2641 | ̶̶ |
| 457 | unnamed1 | CP029000 | 125.913 | 52.298 | ̶̶ |
| 458 | unnamed3 | CP029101 | 208.035 | 52.9247 | ̶̶ |
| 459 | unnamed4 | CP029102 | 135.655 | 54.1528 | ̶̶ |
| 460 | unnamed1 | CP029135 | 70.613 | 51.6151 | ̶̶ |
| 461 | unnamed2 | CP029136 | 190.416 | 52.4594 | ̶̶ |
| 462 | unnamed3 | CP029138 | 81.137 | 53.6426 | ̶̶ |
| 463 | pKp616_1 | CP026496 | 58.11 | 51.7295 | ̶̶ |
| 464 | pKp616_2 | CP026497 | 55.141 | 51.2323 | ̶̶ |
| 465 | unnamed1 | CP028916 | 148.366 | 50.9652 | ̶̶ |
| 466 | pKPC2_095132 | CP028389 | 166.034 | 52.2297 | ̶̶ |
| 467 | pVir_095132 | CP028390 | 208.166 | 49.7074 | ̶̶ |
| 468 | p1-L201 | CP029217 | 288.994 | 56.3787 | ̶̶ |
| 469 | p3-L201 | CP029218 | 102.497 | 50.0863 | ̶̶ |
| 470 | pKPC-L201 | CP029219 | 128.737 | 53.5293 | ̶̶ |
| 471 | p2-L491 | CP029227 | 130.876 | 50.9276 | ̶̶ |
| 472 | pKPC-L491 | CP029230 | 249.533 | 52.034 | ̶̶ |
| 473 | p1-L388 | CP029221 | 217.87 | 49.9431 | ̶̶ |
| 474 | p3-L388 | CP029222 | 142.762 | 59.9683 | ̶̶ |
| 475 | p4-L388 | CP029223 | 87.095 | 53.948 | ̶̶ |
| 476 | pKPC-L388 | CP029225 | 145.851 | 53.7507 | ̶̶ |
| 477 | pKPC2_095649 | CP026584 | 156.099 | 52.9997 | ̶̶ |
| 478 | pDA33140-112 | CP029583 | 112.141 | 51.2801 | ̶̶ |
| 479 | pDA33140-96 | CP029586 | 96.19 | 51.6946 | ̶̶ |
| 480 | pDA33141-217 | CP029588 | 216.772 | 52.6678 | ̶̶ |
| 481 | pDA33144-220 | CP029591 | 219.996 | 52.8214 | ̶̶ |
| 482 | p203 | CP021166 | 177.145 | 51.3506 | ̶̶ |
| 483 | pKpn70742_1 | CP023250 | 65.276 | 53.9402 | ̶̶ |
| 484 | pKpn70742_2 | CP023251 | 63.075 | 51.1914 | ̶̶ |
| 485 | pKpn70747_1 | CP023442 | 186.41 | 53.5508 | ̶̶ |
| 486 | pKpn70747_2 | CP023443 | 117.859 | 54.0756 | ̶̶ |
| 487 | unnamed1 | CP023723 | 297.984 | 46.4431 | + |
| 488 | unnamed2 | CP023724 | 159.072 | 51.6383 | ̶̶ |
| 489 | unnamed3 | CP023725 | 103.454 | 54.1168 | ̶̶ |
| 490 | unnamed1 | CP029739 | 175.746 | 50.268 | ̶̶ |
| 491 | unnamed2 | CP029740 | 103.25 | 53.9128 | ̶̶ |
| 492 | unnamed1 | CP029723 | 183.663 | 52.8936 | ̶̶ |
| 493 | unnamed2 | CP029724 | 178.561 | 51.3791 | ̶̶ |
| 494 | pKPC2_020002 | CP028541 | 177.516 | 53.6729 | ̶̶ |
| 495 | pIMP4_LL34 | CP025964 | 260.97 | 46.6705 | ̶̶ |
| 496 | pNDM1_LL34 | CP025965 | 59.73 | 52.4343 | ̶̶ |
| 497 | pQnrB_LL34 | CP025966 | 130.691 | 52.9547 | ̶̶ |
| 498 | unnamed1 | CP022612 | 334.957 | 47.533 | + |
| 499 | unnamed2 | CP022613 | 102.915 | 50.9848 | ̶̶ |
| 500 | unnamed2 | CP030342 | 113.639 | 53.9287 | ̶̶ |
| 501 | unnamed1 | CP030343 | 207.546 | 52.9039 | ̶̶ |
| 502 | pKpvST101 | CP031369 | 292.735 | 51.6132 | ̶̶ |
| 503 | pKpvST101_5 | CP031372 | 210.779 | 44.7013 | + |
| 504 | pKpvOXA-48 | CP031374 | 65.36 | 51.5055 | ̶̶ |
| 505 | pF1_1 | CP026131 | 164.51 | 53.4594 | ̶̶ |
| 506 | unnamed2 | CP027695 | 177.464 | 51.9164 | ̶̶ |
| 507 | unnamed1 | CP027696 | 207.465 | 52.9786 | ̶̶ |
| 508 | unnamed4 | CP027699 | 71.749 | 51.0934 | ̶̶ |
| 509 | pKJNM10C3.1 | CP030876 | 190.163 | 51.5957 | ̶̶ |
| 510 | pKJNM10C3.2 | CP030878 | 276.504 | 46.3042 | + |
| 511 | p1502320-1 | CP031578 | 60.477 | 49.4915 | ̶̶ |
| 512 | p1502320-3 | CP031580 | 86.8 | 46.5933 | + |
| 513 | pIncFIA-1502320 | CP031581 | 71.335 | 50.3736 | ̶̶ |
| 514 | pIncHI1B-1502320 | CP031582 | 206.229 | 48.6323 | ̶̶ |
| 515 | pIncFII-1502320 | CP031583 | 198.719 | 52.6663 | ̶̶ |
| 516 | pIncAC2-1502320 | CP031584 | 164.082 | 52.4494 | ̶̶ |
| 517 | pIncAC2_L111 | CP030132 | 194.181 | 52.4753 | ̶̶ |
| 518 | pKPC2_L111 | CP030134 | 60.307 | 55.4082 | ̶̶ |
| 519 | pOXA48_L111 | CP030135 | 65.5 | 51.4275 | ̶̶ |
| 520 | unnamed1 | CP031790 | 213.061 | 50.2908 | ̶̶ |
| 521 | unnamed2 | CP031791 | 109.702 | 49.2625 | ̶̶ |
| 522 | unnamed1 | CP031801 | 161.308 | 50.1711 | ̶̶ |
| 523 | unnamed2 | CP031802 | 128.238 | 52.6474 | ̶̶ |
| 524 | unnamed3 | CP031803 | 111.123 | 49.1455 | ̶̶ |
| 525 | unnamed4 | CP031804 | 70.656 | 50.9115 | ̶̶ |
| 526 | unnamed5 | CP031805 | 65.454 | 51.4102 | ̶̶ |
| 527 | unnamed6 | CP031806 | 53.943 | 52.7891 | ̶̶ |
| 528 | unnamed | CP031809 | 233.468 | 51.0524 | ̶̶ |
| 529 | unnamed1 | CP031815 | 139.276 | 49.9433 | ̶̶ |
| 530 | unnamed1 | CP031811 | 169.46 | 52.5888 | ̶̶ |
| 531 | unnamed1 | CP031793 | 141.675 | 49.4929 | ̶̶ |
| 532 | unnamed2 | CP031794 | 53.774 | 53.1074 | ̶̶ |
| 533 | unnamed | CP031799 | 133.343 | 50.0454 | ̶̶ |
| 534 | unnamed1 | CP031796 | 169.772 | 49.0417 | ̶̶ |
| 535 | pZYST1C1 | CP031614 | 196.414 | 51.5671 | ̶̶ |
| 536 | pZYST1C2 | CP031615 | 107.458 | 49.5784 | ̶̶ |
| 537 | pKPC2_020003 | CP031720 | 154.957 | 53.0767 | ̶̶ |
| 538 | pKp1050-1 | CP023417 | 311.641 | 50.2286 | ̶̶ |
| 539 | pKp1050-2 | CP023418 | 138.885 | 55.3868 | ̶̶ |
| 540 | pKp1050-3 | CP023419 | 70.869 | 51.8929 | ̶̶ |
| 541 | pKp1050-4 | CP023420 | 64.94 | 50.6791 | ̶̶ |
| 542 | unnamed | CP023421 | 53.096 | 53.5991 | ̶̶ |
| 543 | pMCR8_095845 | CP031883 | 110.966 | 52.1169 | ̶̶ |
| 544 | pNDM1_095845 | CP031884 | 105.259 | 51.0493 | ̶̶ |
| 545 | pKPC-KP16932 | CM010665 | 158.277 | 53.5378 | ̶̶ |
| 546 | pvir-KP16932 | CM010666 | 177.779 | 50.4593 | ̶̶ |
| 547 | pIncAC2-1502267 | CM010663 | 164.928 | 52.452 | ̶̶ |
| 548 | pIncFIB-1502267 | CM010664 | 201.243 | 52.5911 | ̶̶ |
| 549 | pKp_SE1_2017_2 | CM010661 | 187.517 | 51.6231 | ̶̶ |
| 550 | pKp_SE1_NDM | CM010662 | 75.627 | 52.5368 | ̶̶ |
| 551 | pNDM-KP14003 | CM010660 | 60.951 | 49.2363 | ̶̶ |
| 552 | unnamed1 | CP032168 | 158.987 | 52.564 | ̶̶ |
| 553 | unnamed2 | CP032169 | 171.589 | 52.1187 | ̶̶ |
| 554 | unnamed3 | CP032170 | 65.672 | 52.3008 | ̶̶ |
| 555 | unnamed1 | CP032176 | 90.682 | 53.4748 | ̶̶ |
| 556 | unnamed1 | CP032173 | 153.522 | 51.788 | ̶̶ |
| 557 | unnamed2 | CP032174 | 63.589 | 51.2211 | ̶̶ |
| 558 | unnamed1 | CP032186 | 202.373 | 52.7501 | ̶̶ |
| 559 | unnamed2 | CP032187 | 110.928 | 48.9362 | ̶̶ |
| 560 | unnamed3 | CP032188 | 93.87 | 53.5464 | ̶̶ |
| 561 | unnamed4 | CP032189 | 127.285 | 52.8892 | ̶̶ |
| 562 | unnamed6 | CP032191 | 78.188 | 52.109 | ̶̶ |
| 563 | unnamed1 | CP032195 | 143.515 | 51.2539 | ̶̶ |
| 564 | unnamed2 | CP032196 | 109.398 | 49.351 | ̶̶ |
| 565 | unnamed3 | CP032197 | 73.502 | 53.887 | ̶̶ |
| 566 | unnamed4 | CP032198 | 58.029 | 54.4435 | ̶̶ |
| 567 | unnamed2 | CP032208 | 310.872 | 47.3011 | ̶̶ |
| 568 | unnamed3 | CP032209 | 161.618 | 52.0777 | ̶̶ |
| 569 | unnamed6 | CP032212 | 83.015 | 51.6365 | ̶̶ |
| 570 | unnamed1 | CP032223 | 201.149 | 44.829 | ̶̶ |
| 571 | unnamed2 | CP032224 | 73.056 | 51.0554 | ̶̶ |
| 572 | unnamed3 | CP032225 | 108.879 | 49.3125 | ̶̶ |
| 573 | unnamed4 | CP032226 | 85.41 | 52.7608 | ̶̶ |
| 574 | pKP3301 | AP018748 | 296.75 | 46.5378 | + |
| 575 | pKP3302 | AP018749 | 102.91 | 50.9824 | ̶̶ |
| 576 | pKP6401 | AP018751 | 102.903 | 50.9927 | ̶̶ |
| 577 | pKP6701 | AP018754 | 102.917 | 50.9896 | ̶̶ |
| 578 | pGSU10-3-1 | AP018672 | 159.072 | 51.6383 | ̶̶ |
| 579 | pGSU10-3-2 | AP018673 | 134.879 | 52.7465 | ̶̶ |
| 580 | pGSU10-3-3 | AP018674 | 66.25 | 54.2158 | ̶̶ |
| 581 | unnamed1 | CP031818 | 430.829 | 47.9958 | + |
| 582 | pJYC04A | CP022917 | 246.577 | 52.9798 | ̶̶ |
| 583 | pJYC02A | CP022923 | 246.577 | 52.9798 | ̶̶ |
| 584 | pJYC03A | CP022920 | 246.577 | 52.9798 | ̶̶ |
| 585 | pJYC01A | CP022926 | 258.21 | 52.9352 | ̶̶ |
| 586 | p1_020049 | CP028784 | 182.097 | 52.1585 | ̶̶ |
| 587 | pNDM1_020049 | CP028786 | 54.035 | 49.0349 | ̶̶ |
| 588 | pINF078-VP | CP032832 | 399.913 | 53.2138 | ̶̶ |
| 589 | pINF237_01-VP | CP032834 | 133.713 | 50.2038 | ̶̶ |
| 590 | pINF237_02 | CP032835 | 93.621 | 51.5568 | ̶̶ |
| 591 | pINF237_03 | CP032836 | 76.772 | 50.62 | ̶̶ |
| 592 | unnamed1 | CP028479 | 129.864 | 50.5775 | ̶̶ |
| 593 | unnamed2 | CP028480 | 77.91 | 54.1278 | ̶̶ |
| 594 | p1_115069 | CP033402 | 208.288 | 52.5657 | ̶̶ |
| 595 | pKPC2_115069 | CP033404 | 154.986 | 53.0429 | ̶̶ |
| 596 | pCTXM65_015625 | CP033394 | 142.207 | 53.2076 | ̶̶ |
| 597 | pKPC12_015625 | CP033395 | 55.373 | 55.0593 | ̶̶ |
| 598 | unnamed1 | CP033755 | 103.852 | 52.0934 | ̶̶ |
| 599 | unnamed2 | CP033758 | 232.752 | 50.9362 | ̶̶ |
| 600 | unnamed1 | CP033774 | 184.336 | 52.7097 | ̶̶ |
| 601 | unnamed2 | CP033775 | 74.543 | 54.5953 | ̶̶ |
| 602 | unnamed3 | CP033776 | 73.896 | 50.1908 | ̶̶ |
| 603 | pNJST258N2 | CP006926 | 73.636 | 53.9369 | ̶̶ |
| 604 | pNJST258N1 | CP006927 | 142.788 | 51.7761 | ̶̶ |
| 605 | p30684_2b | CP006922 | 86.232 | 54.5668 | ̶̶ |
| 606 | p500_1420-130.552kb | CP011981 | 130.552 | 54.0643 | ̶̶ |
| 607 | p500_1420-51.662kb | CP011982 | 51.662 | 53.8074 | ̶̶ |
| 608 | pDMC1097-218.836kb | CP011977 | 218.836 | 52.7633 | ̶̶ |
| 609 | pDMC1097-77.775kb | CP011978 | 77.775 | 45.4131 | ̶̶ |
| 610 | pKPC_FCF13/05 | CP004366 | 53.081 | 52.5499 | ̶̶ |
| 611 | pKPC_FCF/3SP | CP004367 | 54.605 | 52.9127 | ̶̶ |
| 612 | p1 | CP006657 | 317.154 | 53.0455 | ̶̶ |
| 613 | pKCTC2242 | CP002911 | 202.852 | 50.1696 | ̶̶ |
| 614 | pKP1-19 | CP012884 | 194.742 | 51.9508 | ̶̶ |
| 615 | pK45-67VIM | HF955507 | 56.171 | 51.8631 | ̶̶ |
| 616 | unnamed1 | CP027613 | 191.856 | 51.5892 | ̶̶ |
| 617 | unnamed2 | CP027614 | 100.759 | 55.9087 | ̶̶ |
| 618 | unnamed3 | CP027615 | 108.623 | 49.3275 | ̶̶ |
| 619 | unnamed4 | CP027616 | 129.985 | 54.147 | ̶̶ |
| 620 | unnamed5 | CP027617 | 54.242 | 51.7588 | ̶̶ |
| 621 | pKPC-63d | CP009773 | 75.618 | 54.0162 | ̶̶ |
| 622 | pKPC-def | CP009776 | 115.32 | 51.2496 | ̶̶ |
| 623 | pKPN-a68 | CP009777 | 212.192 | 52.7975 | ̶̶ |
| 624 | pKPC-e4e | CP009864 | 62.589 | 53.0333 | ̶̶ |
| 625 | pKPN-80a | CP009865 | 159.36 | 51.4163 | ̶̶ |
| 626 | pKPN-b9c | CP009874 | 50.051 | 51.3456 | ̶̶ |
| 627 | pKpQIL-531 | CP009875 | 113.639 | 53.9287 | ̶̶ |
| 628 | pKPN-852 | CP009878 | 51.622 | 53.4908 | ̶̶ |
| 629 | pKPN-c22 | CP009879 | 178.563 | 51.5185 | ̶̶ |
| 630 | pKpn23412-362 | CP011314 | 361.964 | 47.9981 | + |
| 631 | unnamed1 | CP014005 | 101.03 | 51.7104 | ̶̶ |
| 632 | pRJF999 | CP014011 | 228.907 | 50.0968 | ̶̶ |
| 633 | pRJF293 | CP014009 | 224.263 | 50.1166 | ̶̶ |
| 634 | p1 | CP019773 | 208.528 | 52.9325 | ̶̶ |
| 635 | p2 | CP019774 | 114.073 | 53.8804 | ̶̶ |
| 636 | pKpvST147L | CM007852 | 343.282 | 46.9203 | + |
| 637 | pRJA166a | CP019048 | 230.606 | 45.8371 | + |
| 638 | pRJA166b | CP019049 | 228.613 | 50.0496 | ̶̶ |
| 639 | pRJA166c | CP019050 | 111.083 | 49.0066 | ̶̶ |
| 640 | pAUSMDU8079-1 | CP022692 | 207.349 | 52.9359 | ̶̶ |
| 641 | pAUSMDU8079-2 | CP022693 | 113.639 | 53.9304 | ̶̶ |
| 642 | p18-43_01 | CP023554 | 212.326 | 53.7028 | ̶̶ |
| 643 | p18-43_02 | CP023555 | 169.145 | 53.8786 | ̶̶ |
| 644 | p18-43_03 | CP023556 | 65.209 | 52.6415 | ̶̶ |
| 645 | p19-10_01 | CP023488 | 223.434 | 51.4483 | ̶̶ |
| 646 | p19-10_02 | CP023489 | 124.323 | 52.4915 | ̶̶ |
| 647 | pKPGD4 | CP025952 | 170.821 | 53.4683 | ̶̶ |
| 648 | unnamed1 | CP018884 | 121.863 | 51.4036 | ̶̶ |
| 649 | pIncF | CP018886 | 217.456 | 52.4984 | ̶̶ |
| 650 | pIncN | CP018887 | 123.208 | 52.7547 | ̶̶ |
| 651 | pKpvK54 | CP023135 | 211.454 | 49.624 | ̶̶ |
| 652 | pNDM5_020046 | CP028781 | 159.394 | 51.0452 | ̶̶ |
| 653 | pQnrB52_020046 | CP028782 | 74.017 | 51.6895 | ̶̶ |
| 654 | p1_020143 | CP028543 | 159.355 | 52.2393 | ̶̶ |
| 655 | p2_020143 | CP028544 | 110.562 | 48.8866 | ̶̶ |
| 656 | pKPC2_020143 | CP028547 | 73.791 | 54.8414 | ̶̶ |
| 657 | pKPC2_020079 | CP029381 | 146.79 | 53.5493 | ̶̶ |
| 658 | pQnrS1_020079 | CP029382 | 85.185 | 53.8487 | ̶̶ |
| 659 | pVir_020079 | CP029383 | 178.871 | 49.3736 | ̶̶ |
| 660 | pNDM6_040074 | CP029386 | 52.989 | 49.2838 | ̶̶ |
| 661 | pTetD_040074 | CP029387 | 226.524 | 52.2828 | ̶̶ |
| 662 | pSC7-IncFIB-110K | CP030268 | 111.007 | 48.9284 | ̶̶ |
| 663 | pSC7-vir | CP030270 | 236.809 | 49.996 | ̶̶ |
| 664 | pKC-Pl-HB1 | CP030924 | 196 | 50.5332 | ̶̶ |
| 665 | pKJNM8C2.1 | CP030858 | 304.592 | 47.756 | + |
| 666 | pKJNM8C2.2 | CP030859 | 457.831 | 50.4667 | ̶̶ |
| 667 | pKPM501 | CP031735 | 253.531 | 51.2233 | + |
| 668 | pKPM502 | CP031736 | 250.351 | 46.3925 | ̶̶ |
| 669 | pKPHS1 | CP003223 | 122.799 | 49.4613 | ̶̶ |
| 670 | pKPHS3 | CP003225 | 105.974 | 52.4591 | ̶̶ |
| 671 | pKPHS2 | CP003224 | 111.195 | 53.3055 | ̶̶ |
| 672 | pKP13e | CP003998 | 81.071 | 51.1169 | ̶̶ |
| 673 | pKP13f | CP004000 | 295.493 | 47.9453 | ̶̶ |
| 674 | pKPN-498 | CP008829 | 243.824 | 53.2249 | ̶̶ |
| 675 | pKpQIL-6e6 | CP008830 | 113.639 | 53.9278 | ̶̶ |
| 676 | pKPN-498 | CP007729 | 243.824 | 53.2249 | ̶̶ |
| 677 | pKpQIL-6e6 | CP007730 | 113.639 | 53.9278 | ̶̶ |
| 678 | pKPC-484 | CP008798 | 85.473 | 55.579 | ̶̶ |
| 679 | pKPN-e44 | CP008800 | 194.877 | 52.47 | ̶̶ |
| 680 | pKEC-dc3 | CP007732 | 268.334 | 52.6139 | ̶̶ |
| 681 | pKPN-068 | CP007733 | 80.411 | 51.3437 | ̶̶ |
| 682 | pKPN-262 | CP007734 | 338.85 | 52.721 | ̶̶ |
| 683 | pKPN-a41 | CP007735 | 89.77 | 51.818 | ̶̶ |
| 684 | pKPN-b0b | CP007736 | 113.44 | 49.3926 | ̶̶ |
| 685 | pKpQIL-531 | CP008833 | 113.639 | 53.9287 | ̶̶ |
| 686 | pKPX-1 | AP012055 | 250.444 | 53.4207 | ̶̶ |
| 687 | pKPX-2 | AP012056 | 141.545 | 52.4928 | ̶̶ |
| 688 | pKPN3 | CP000648 | 175.879 | 51.6907 | ̶̶ |
| 689 | pKPN4 | CP000649 | 107.576 | 53.4404 | ̶̶ |
| 690 | pKPN5 | CP000650 | 88.582 | 53.8168 | ̶̶ |
| 691 | pK2044 | AP006726 | 224.152 | 50.1682 | ̶̶ |
| 692 | plasmid1 | CP006799 | 283.371 | 46.4105 | + |
| 693 | plasmid2 | CP006800 | 103.694 | 50.9962 | ̶̶ |
| 694 | plasmid3 | CP006801 | 70.814 | 52.9217 | ̶̶ |
| 695 | pKRH | FO203500 | 113.685 | 52.6956 | ̶̶ |
| 696 | pUHKPC07-113.639kb | CP011986 | 113.639 | 53.9251 | ̶̶ |
| 697 | pUHKPC07-74.026kb | CP011987 | 74.026 | 50.412 | ̶̶ |
| 698 | pUHKPC33-162.533kb | CP011990 | 162.533 | 53.73 | ̶̶ |
| 699 | pUHKPC33-113.638kb | CP011991 | 113.638 | 53.9283 | ̶̶ |

^a^ +, presence; -, absent

**Table S2**. **List of unique spacers found in the CRISPR-array of plasmids and their match with protospacers**

| Spacers | Sequences | Protospacer match**^a, b^** |
| --- | --- | --- |
| SP1 | AGCAACGTTTCCGGATTATATGGCTGGAACGT | Plasmid CRISPR-array |
| SP2 | TGGTGCTCTCAACCGTCACCCGCTGGCTGGAA | Plasmid CRISPR-array |
| SP3 | AGGTATTTGACCTCATCCAGAAAGGCACAGAC | Plasmid CRISPR-array |
| SP4 | GCACCCTCACGGATACCTTTTGCACAGTGTTA | Plasmid CRISPR-array |
| SP5 | CTTAGAGAAGCAAAAACCCCACCGAGGCAGGG | Plasmid CRISPR-array |
| SP6 | TTACTTTTTGGCAGTTGGTAAAACACTTTTGC | Plasmid CRISPR-array |
| SP7 | CGAAAACGGCAACCTTCATAAAAACGTCTTTT | Plasmid CRISPR-array |
| SP8 | CCGAGATTGAGTAAAGCAAAGTAACGGCGGTG | *Kp* chromosome |
| SP9 | TGTGTGTTGGCGTTCGTTAAATATTGTTAGTA | Plasmid CRISPR-array |
| SP10 | GCTGGATTTCCGTCAGTTGGTCAGCTGCTGCT | Plasmid CRISPR-array |
| SP11 | TTCCGGACTCCTGTTTCCGGCAGTGGATTAAA | Plasmid *traL* gene |
| SP12 | CACTACCAGATCCGAATGGACACCCGTAATGA | Plasmid CRISPR-array |
| SP13 | TGGTGTTGTCCACGGTTACCCGCTGGCTGGAA | Plasmid CRISPR-array |
| SP14 | TCGTGTTGTCCACGGTTACCCGCTGGCTGGAA | Plasmid CRISPR-array |
| SP15 | CAGGGTTTGCCCTTTTGCACCGCGCAGCGGTC | Plasmid CRISPR-array |
| SP16 | CGTTCTATCGGATAATACATTTTGTGATTTTA | No match |
| SP17 | GATGGTCTTCCAAATATGGCCTATAGCGAAAC | No match |
| SP18 | GAGTGAAAGTGATAGCAAGAGTGTAATGGCTA | Plasmid CRISPR-array |
| SP19 | AATCAATACGCCCTGATGCTTAAAAATGGCCG | Plasmid CRISPR-array |
| SP20 | TCCGAGCTACCGATTTACCAGGAGAGCGCTCG | *Kp* chromosome |
| SP21 | CAACAGGGATGTTTCGTCCGTTCAGACGCTG | Plasmid CRISPR-array |
| SP22 | TTCCAGCCAGCGGGTGACCGTGGACAACACCA | Plasmid CRISPR-array |
| SP23 | TTTCCTTAGACAGCCATTAGGAATTGAGGTTG | Plasmid CRISPR-array |
| SP24 | AATACGCCAAACGGGCATTTGAGTCGCGCCAGCCCAT | Plasmid CRISPR-array |
| SP25 | TATTTTATTGACTGGTTAGATATCTCTCAGGA | No match |
| SP26 | TACAAAGACATTTGTTGTCCAGGGTGACAGTA | No match |
| SP27 | ACCACACTCTCGCAGTGGTTCTCGCTCATGCC | Plasmid CRISPR-array |
| SP28 | TATTCAGCCAGTTCTCCTGGGCACCGGGTCGG | Plasmid CRISPR-array |
| SP29 | GAACAGGTCCCAATTCTCCGTCAGCAGCCTCA | Plasmid CRISPR-array |
| SP30 | CAGCAGGTGCGCGTCAGAGTGCGCAGGCTCATG | Plasmid CRISPR-array |
| SP31 | CCATTTTTCAAACAGCCGGATTTTCGATTTGCT | Plasmid CRISPR-array |
| SP32 | CATCACCACACTCATAAATTAACAAAACATTAA | Plasmid CRISPR-array |
| SP33 | CTGCACTGCCTGTCACTATTCCTCGAACTGCT | Plasmid CRISPR-array |
| SP34 | GAAACGACCTGGCGCAGAAACTTCAAATTGTT | Plasmid CRISPR-array |
| SP35 | CCTGACGACATCGGATCACATTTGCGTTACCG | Plasmid CRISPR-array |
| SP36 | ATTTACAAATGAAGATTTTTCCCCATTGGTAA | Plasmid intergenic region at *tra* region |
| SP37 | GCACCCTCACGTATACCTTTTGCACAGTGTTA | Plasmid CRISPR-array |
| SP38 | AACATCAGTGGAAATCCACTGCGGC | Plasmid CRISPR-array |
| SP39 | CAGGTTATACTGGCAAAACGTCGATGGCTCT | Plasmid CRISPR-array |
| SP40 | TAAATCAGCAAATATTGTTGTCTACCGTGTCG | Plasmid CRISPR-array |
| SP41 | TGAGGCTGCTGACGGAGAAATGGGACCTGTTC | Plasmid CRISPR-array |
| SP42 | TTCCCTGCACTAAGACGCTGGTGGTCGCCAC | *Klebsiella* chromosome |
| SP43 | CCGGTTCGGATTTTGCGAAACAGGTGCAGGGC | Plasmid *traN* gene |
| SP44 | CCCTGCCTCGGTGGTTTTTTTGCTTCTCTAAG | No match |
| SP45 | GTACAGGTTCATCAGGATCCGGGGTAAAGTT | Plasmid CRISPR-array |
| SP46 | GCTATAGAACTGAGCTACACCACGGGCCGCAC | No match |
| SP47 | GGAATGTCCTTGATTATATAAAGGCTGTCAGT | No match |
| SP48 | TGACTTTTAAAAAAAATCAGCTGAAACAGCC | No match |
| SP49 | ATCAGCCTGCGGGGTTCGGAGCTGCGTGTCTC | No match |
| SP50 | GAAACTATTTCGGAGCATCTCATTGCTGAGG | No match |
| SP51 | TTCCAGCCAGCGGGTAACGGTTGAGAGCACCA | No match |
| SP52 | GAAGAGCGACAATAAAACTGGCTTGTCTTTTC | No match |
| SP53 | TGTGCGGGGGTTATCGGTCGTGTTGTCCACGGTT | Plasmid CRISPR-array |
| SP54 | CCTTAGAGAAGCAAAACCCCACCGAGGCAGGG | No match |
| SP55 | TTCTCTCCGCCGGGCAGTGTGATGCCGGAGGGGTATTC | Plasmid transcriptional regulator/*repC-*like gene |
| SP56 | TAAATCAGCAAATCTTGTTGTCTACCGTGTCGGTATTC | Plasmid CRISPR-array |
| SP57 | AAGTTATTCATGTCGCCATTCACGTCGGCGGCGTATTT | Plasmid *traH* gene |
| SP58 | TTACGCATTGTGATCAGGATTCGCTTCAGACGGTATTC | Plasmid CRISPR-array |
| SP59 | AATGGTGACAGGCAGTGCACCGGCCTGGAGGAGTATTA | Plasmid CRISPR-array |
| SP60 | TCGCCCAGTCCAAAGGGGATGATGCAGGATTGGTATTC | Plasmid CRISPR-array |
| SP61 | TACTACCAATATTTAACGAACGTCGTCAGACA | No match |
| SP62 | TTGGCGACCACCAGCGTTTTAGTGCAGGGAAC | *Klebsiella* *spp.* chromosome |
| SP63 | AGTTTGTATGAAAGCCTCATGTTTTGCACCTGTGCCGGTGCATATCATCCTCAGAGC | *Klebsiella* *spp*. chromosome |
| SP64 | ATTTTGCGCCACAGAGGTGGTTCGATATCAAA | No match |
| SP65 | ATACCTCTGTCAATGCCCTCGTTGAACGTTTT | No match |
| SP66 | CGTTACCGCTCATAAGATCTCCTGAGTTTTGT | No match |
| SP67 | GGTTGAATTTGTAAAAACCTATGCTAATAATT | No match |

**^a^** The term “Plasmid CRISPR-array” representing spacer had match with other plasmid CRISPR regions (spacers or repeats) only and there is no match with other plasmids and chromosomes in the GenBank.

**^b^** “No match” means, no match was identified in the GenBank except this spacer

**Table S3. List of *K. pneumoniae* strains analysed for chromosomal CRISPR-Cas system**

| **Sl. No.** | **Strain ID** | **Accession no.** | **Type of CRISPR-Cas** | **No. of plasmids** | **No. of spacers** | **Plasmid CRISPR-Cas^a^** |
| --- | --- | --- | --- | --- | --- | --- |
| 1 | HS11286 | CP003200.1 | 0 | 6 |  | ˗ |
| 2 | NTUH-K2044 | AP006725.1 | I-E* | 1 | 28 | ˗ |
| 3 | ATCC 700721; MGH 78578 | CP000647.1 | 0 | 5 |  | ˗ |
| 4 | KCTC 2242 | CP002910.1 | 0 | 1 |  | ˗ |
| 5 | KPNIH10 | CP007727.1 | 0 | 3 |  | ˗ |
| 6 | KPNIH1 | CP008827.1 | 0 | 3 |  | ˗ |
| 7 | 1084 | CP003785.1 | I-E* | 0 | 14 | ˗ |
| 8 | ATCC BAA-2146 | CP006659.2 | 0 | 4 |  | ˗ |
| 9 | 500_1420 | CP011980.1 | 0 | 4 |  | ˗ |
| 10 | UHKPC33 | CP011989.1 | 0 | 4 |  | ˗ |
| 11 | DMC1097 | CP011976.1 | 0 | 3 |  | ˗ |
| 12 | UHKPC07 | CP011985.1 | 0 | 3 |  | ˗ |
| 13 | JM45 | CP006656.1 | 0 | 2 |  | ˗ |
| 14 | KP-1 | CP012883.1 | 0 | 1 |  | ˗ |
| 15 | CG43 | CP006648.1 | 0 | 0 |  | ˗ |
| 16 | Kp13 | CP003999.1 | 0 | 6 |  | ˗ |
| 17 | 30684/NJST258_2 | CP006918.1 | 0 | 3 |  | ˗ |
| 18 | 30660/NJST258_1 | CP006923.1 | 0 | 5 |  | ˗ |
| 19 | KPNIH27 | CP007731.1 | I-E | 5 | 39 | ˗ |
| 20 | KPNIH24 | CP008797.1 | 0 | 3 |  | ˗ |
| 21 | KPR0928 | CP008831.1 | 0 | 2 |  | ˗ |
| 22 | PittNDM01 | CP006798.1 | I-E* | 4 | 21 | + |
| 23 | blaNDM-1 | CP009114.1 | 0 | 2 |  | ˗ |
| 24 | ATCC 43816 KPPR1 | CP009208.1 | I-E* | 0 | 17 | ˗ |
| 25 | PMK1 | CP008929.1 | I-E* | 4 | 17 | **+** |
| 26 | KPNIH33 | CP009771.1 | 0 | 3 |  | ˗ |
| 27 | KPNIH32 | CP009775.1 | 0 | 3 |  | ˗ |
| 28 | XH209 | CP009461.1 | 0 | 0 |  | ˗ |
| 29 | KPNIH29 | CP009863.1 | I-E | 2 | 3 | ˗ |
| 30 | KPNIH30 | CP009872.1 | 0 | 3 |  | ˗ |
| 31 | KPNIH31 | CP009876.1 | I-E | 3 | 39 | ˗ |
| 32 | 32192 | CP010361.1 | 0 | 3 |  | ˗ |
| 33 | HK787 | CP006738.1 | 0 | 0 |  | ˗ |
| 34 | 34618 | CP010392.1 | 0 | 4 |  | ˗ |
| 35 | 1158 | CP006722.1 | 0 | 0 |  | ˗ |
| 36 | Kp52.145 | FO834906.1 | I-E | 2 | 18 | ˗ |
| 37 | 234-12 | CP011313.1 | 0 | 3 |  | ˗ |
| 38 | CAV1392 | CP011578.1 | 0 | 3 |  | ˗ |
| 39 | CAV1344 | CP011624.1 | I-E | 5 | 45 | ˗ |
| 40 | CAV1596 | CP011647.1 | 0 | 4 |  | ˗ |
| 41 | KP617 | CP012753.1 | I-E* | 2 | 12 | **+** |
| 42 | ST101:960186733 | CP023487.1 | 0 | 2 |  | ˗ |
| 43 | MS6671 | LN824133.1 | I-E | 6 | 43 | **+** |
| 44 | KpN01 | CP012987.1 | 0 | 4 |  | ˗ |
| 45 | KpN06 | CP012992.1 | 0 | 4 |  | ˗ |
| 46 | CAV1193 | CP013322.1 | I-E | 5 | 42 | ˗ |
| 47 | J1 | CP013711.1 | I-E* | 2 | 15 | ˗ |
| 48 | NUHL24835 | CP014004.1 | I-E* | 2 | 9 | ˗ |
| 49 | RJF999 | CP014010.1 | I-E* | 1 | 27 | **+** |
| 50 | RJF293 | CP014008.1 | I-E* | 1 | 8 | ˗ |
| 51 | YH43 | AP014950.1 | I-E | 0 | 11 | ˗ |
| 52 | TGH8 | CP012743.1 | I-E | 0 | 24 | ˗ |
| 53 | TGH10 | CP012744.1 | I-E | 0 | 24 | ˗ |
| 54 | SKGH01 | CP015500.1 | I-E | 5 | 43 | **+** |
| 55 | W14 | CP015753.1 | 0 | 2 |  | ˗ |
| 56 | AATZP | CP014755.1 | I-E | 3 | 43 | ˗ |
| 57 | BR | CP015990.1 | I-E* | 1 | 11 | ˗ |
| 58 | KPNIH39 | CP014762.1 | 0 | 3 |  | ˗ |
| 59 | Kpn223 | CP015025.1 | I-E | 1 | 43 | ˗ |
| 60 | Kpn555 | CP015130.1 | 0 | 3 |  | ˗ |
| 61 | KPNIH36 | CP014647.1 | 0 | 3 |  | ˗ |
| 62 | TH1 | CP016159.1 | 0 | 2 |  | ˗ |
| 63 | DHQP1002001 | CP016811.1 | 0 | 2 |  | ˗ |
| 64 | blood sample 2 | CP015822.1 | 0 | 3 |  | ˗ |
| 65 | ED23 | CP016814.1 | I-E* | 1 | 21 | ˗ |
| 66 | ED2 | CP016813.1 | I-E* | 0 | 19 | ˗ |
| 67 | 11 | CP016923.1 | I-E* | 4 | 9 | **+** |
| 68 | 23 | CP016926.1 | I-E* | 3 | 20 | ˗ |
| 69 | UCLAOXA232KP_Pt0 | CP012560.1 | 0 | 0 |  | ˗ |
| 70 | UCLAOXA232KP | CP012568.1 | 0 | 4 |  | ˗ |
| 71 | UCLAOXA232KP | CP012561.1 | 0 | 6 |  | ˗ |
| 72 | TGH13 | CP012745.1 | I-E | 0 | 41 | ˗ |
| 73 | KP36 | CP017385.1 | I-E* | 3 | 20 | ˗ |
| 74 | KP5 | CP012426.1 | I-E | 4 | 43 | ˗ |
| 75 | CAV1016 | CP017934.1 | I-E | 3 | 64 | ˗ |
| 76 | Kp_Goe_822579 | CP018140.1 | I-E | 6 | 35 | **+** |
| 77 | MNCRE78 | CP018428.1 | 0 | 4 |  | ˗ |
| 78 | MNCRE69 | CP018427.1 | 0 | 4 |  | ˗ |
| 79 | MNCRE53 | CP018437.1 | 0 | 4 |  | ˗ |
| 80 | CAV1453 | CP018356.1 | 0 | 3 |  | ˗ |
| 81 | Kp_Goe_154414 | CP018337.1 | 0 | 6 |  | ˗ |
| 82 | Kp_Goe_62629 | CP018364.1 | 0 | 2 |  | ˗ |
| 83 | Kp_Goe_39795 | CP018458.1 | I-E* | 4 | 20 | ˗ |
| 84 | Kp_Goe_822917 | CP018438.1 | 0 | 8 |  | ˗ |
| 85 | Kp_Goe_33208 | CP018447.1 | 0 | 2 |  | ˗ |
| 86 | SWU01 | CP018454.1 | 0 | 1 |  | ˗ |
| 87 | Kp_Goe_71070 | CP018450.1 | 0 | 3 |  | ˗ |
| 88 | CAV1417 | CP018352.1 | 0 | 4 |  | ˗ |
| 89 | CAV1042 | CP018671.1 | 0 | 6 |  | ˗ |
| 90 | Kp_Goe_149473 | CP018686.1 | I-E | 5 | 35 | **+** |
| 91 | Kp_Goe_827024 | CP018701.1 | I-E | 5 | 35 | **+** |
| 92 | Kp_Goe_827026 | CP018707.1 | I-E | 5 | 35 | **+** |
| 93 | Kp_Goe_152021 | CP018713.1 | I-E | 5 | 35 | **+** |
| 94 | KP_Goe_828304 | CP018719.1 | I-E | 5 | 35 | **+** |
| 95 | CAV1217 | CP018676.1 | 0 | 4 |  | ˗ |
| 96 | Kp_Goe_149832 | CP018695.1 | I-E | 5 | 35 | **+** |
| 97 | Kp_Goe_821588 | CP018692.1 | 0 | 2 |  | ˗ |
| 98 | Kp_Goe_121641 | CP018735.1 | 0 | 5 |  | ˗ |
| 99 | AR_0049 | CP018816.1 | 0 | 3 |  | ˗ |
| 100 | ATCC 35657 | CP015134.1 | I-E* | 1 | 26 | ˗ |
| 101 | 1756 | CP019219.1 | 0 | 1 |  | ˗ |
| 102 | CN1 | CP015382.1 | I-E | 2 | 39 | ˗ |
| 103 | NY9 | CP015385.1 | 0 | 6 |  | ˗ |
| 104 | CR14 | CP015392.1 | 0 | 5 |  | ˗ |
| 105 | 825795-1 | CP017985.1 | I-E | 4 | 35 | **+** |
| 106 | KP38731 | CP014294.1 | 0 | 6 |  | ˗ |
| 107 | AR_0117 | CP020061.1 | I-E* | 5 | 14 | ˗ |
| 108 | AR_0068 | CP020067.1 | I-E* | 3 | 21 | **+** |
| 109 | AR_0115 | CP020071.1 | 0 | 4 |  | ˗ |
| 110 | AR_0098 | CP020108.1 | 0 | 4 |  | ˗ |
| 111 | K66-45 | CP020901.1 | 0 | 4 |  | ˗ |
| 112 | kp757 | CP015120.1 | 0 | 0 |  | ˗ |
| 113 | BK13043 | CP020837.1 | 0 | 3 |  | ˗ |
| 114 | KPN1482 | CP020841.1 | 0 | 5 |  | ˗ |
| 115 | KPN528 | CP020853.1 | I-E* | 3 | 21 | **+** |
| 116 | RJA166 | CP019047.1 | I-E* | 3 | 40 | **+** |
| 117 | AR_0047 | CP021539.1 | 0 | 4 |  | ˗ |
| 118 | AR_0112 | CP021549.1 | 0 | 5 |  | ˗ |
| 119 | AR_0146 | CP021685.1 | 0 | 3 |  | ˗ |
| 120 | AR_0158 | CP021696.1 | I-E | 4 | 52 | **+** |
| 121 | AR_0129 | CP021718.1 | 0 | 5 |  | ˗ |
| 122 | AR_0143 | CP021708.1 | I-E* | 4 | 21 | ˗ |
| 123 | AR_0126 | CP021740.1 | I-E | 3 | 64 | ˗ |
| 124 | AR_0113 | CP021751.1 | 0 | 5 |  | ˗ |
| 125 | AR_0138 | CP021757.1 | I-E | 4 | 43 | ˗ |
| 126 | AR_0120 | CP021833.1 | 0 | 4 |  | ˗ |
| 127 | AR_0125 | CP021859.1 | 0 | 5 |  | ˗ |
| 128 | AR_0139 | CP021960.1 | 0 | 4 |  | ˗ |
| 129 | AR_0145 | CP021939.1 | I-E | 4 | 43 | ˗ |
| 130 | AR_0152 | CP021944.1 | I-E | 5 | 43 | ˗ |
| 131 | AR_0148 | CP021950.1 | 0 | 4 |  | ˗ |
| 132 | AR_0107 | CP021955.1 | 0 | 2 |  | ˗ |
| 133 | 19051 | CP022023.1 | I-E* | 0 | 19 | ˗ |
| 134 | DHQP1605752_NV | CP022127.1 | I-E | 3 | 17 | ˗ |
| 135 | BIC-1 | CP022573.1 | 0 | 3 |  | ˗ |
| 136 | AUSMDU00008079 | CP022691.1 | 0 | 3 |  | ˗ |
| 137 | 911021 | CP022882.1 | 0 | 0 |  | ˗ |
| 138 | 721005 | CP022997.1 | 0 | 0 |  | ˗ |
| 139 | FDAARGOS_445 | CP023502.1 | 0 | 1 |  | ˗ |
| 140 | FDAARGOS_444 | CP023941.1 | 0 | 4 |  | ˗ |
| 141 | FDAARGOS_446 | CP023946.1 | 0 | 3 |  | ˗ |
| 142 | FDAARGOS_439 | CP023913.1 | I-E | 5 | 43 | ˗ |
| 143 | KSB1_5D | CP024191.1 | 0 | 6 |  | ˗ |
| 144 | INF249 | CP024489.1 | 0 | 6 |  | ˗ |
| 145 | INF322 | CP024482.1 | 0 | 6 |  | ˗ |
| 146 | KSB1_7E | CP024496.1 | 0 | 2 |  | ˗ |
| 147 | QS17-0161 | CP024458.1 | I-E | 7 | 62 | ˗ |
| 148 | INF278 | CP024563.1 | 0 | 6 |  | ˗ |
| 149 | INF042 | CP024542.1 | 0 | 2 |  | ˗ |
| 150 | KSB2_1B | CP024504.1 | 0 | 10 |  | ˗ |
| 151 | KSB1_10J | CP024515.1 | 0 | 5 |  | ˗ |
| 152 | INF158 | CP024521.1 | 0 | 6 |  | ˗ |
| 153 | INF157 | CP024528.1 | 0 | 6 |  | ˗ |
| 154 | KSB1_9D | CP024535.1 | 0 | 6 |  | ˗ |
| 155 | INF059 | CP024545.1 | 0 | 2 |  | ˗ |
| 156 | KSB1_7J | CP024548.1 | 0 | 0 |  | ˗ |
| 157 | INF163 | CP024549.1 | 0 | 6 |  | ˗ |
| 158 | INF164 | CP024556.1 | 0 | 6 |  | ˗ |
| 159 | INF274 | CP024570.1 | 0 | 5 |  | ˗ |
| 160 | KSB1_4E | CP024499.1 | 0 | 4 |  | ˗ |
| 161 | P1428 | CP017994.1 | I-E* | 0 | 19 | ˗ |
| 162 | H11 | CP018056.1 | 0 | 0 |  | ˗ |
| 163 | CRKP-1215 | CP024838.1 | I-E | 3 | 43 | ˗ |
| 164 | CRKP-2297 | CP024834.1 | I-E | 3 | 43 | ˗ |
| 165 | WCHKP34 | CP025963.2 | I-E | 3 | 42 | ˗ |
| 166 | AUSMDU00003562 | CP025005.1 | 0 | 2 |  | ˗ |
| 167 | AUSMDU00008119 | CP025008.1 | 0 | 2 |  | ˗ |
| 168 | NU-CRE047 | CP025037.1 | 0 | 5 |  | ˗ |
| 169 | SGH10 | CP025080.1 | I-E* | 1 | 22 | ˗ |
| 170 | 459 | CP018306.1 | 0 | 0 |  | ˗ |
| 171 | NR5632 | CP025143.1 | 0 | 2 |  | ˗ |
| 172 | KP1768 | CP025140.1 | 0 | 2 |  | ˗ |
| 173 | KP1766 | CP025146.1 | 0 | 2 |  | ˗ |
| 174 | KP69 | CP025456.1 | 0 | 4 |  | ˗ |
| 175 | JS187 | CP025466.1 | 0 | 4 |  | ˗ |
| 176 | F44 | CP025461.1 | 0 | 4 |  | ˗ |
| 177 | GD4 | CP025951.1 | 0 | 1 |  | ˗ |
| 178 | KPNIH50 | CP026177.1 | 0 | 5 |  | ˗ |
| 179 | KPNIH49 | CP026178.1 | 0 | 8 |  | ˗ |
| 180 | KPNIH48 | CP026392.1 | 0 | 6 |  | ˗ |
| 181 | NUHL30457 | CP026586.1 | 0 | 4 |  | ˗ |
| 182 | AR_0066 | CP026751.1 | I-E* | 2 | 21 | ˗ |
| 183 | BR7 | CP018883.1 | 0 | 1 |  | ˗ |
| 184 | BR21 | CP018885.1 | 0 | 2 |  | ˗ |
| 185 | FDAARGOS_156 | CP014123.1 | I-E* | 4 | 25 | ˗ |
| 186 | SB3432 | FO203501.1 | 0 | 1 |  | ˗ |
| 187 | ST2017:950142398 | CP023553.1 | 0 | 4 |  | ˗ |
| 188 | U25 | CP012043.1 | I-E* | 0 | 21 | ˗ |
| 189 | KPN_KPC_HUG_07 | CP019772.1 | 0 | 4 |  | ˗ |
| 190 | 704SK6 | CP022143.1 | 0 | 4 |  | ˗ |
| 191 | KP6 | CP025087.1 | I-E* | 0 | 56 | ˗ |
| 192 | KP9 | CP025090.1 | I-E* | 0 | 36 | ˗ |
| 193 | KP10 | CP025091.1 | 0 | 0 |  | ˗ |
| 194 | KP14 | CP025093.1 | 0 | 0 |  | ˗ |
| 195 | KP7 | CP025088.1 | I-E* | 0 | 22 | ˗ |
| 196 | LS357 | CP025639.1 | I-E* | 1 | 27 | **+** |
| 197 | HS09565 | CP025631.1 | I-E* | 1 | 28 | **+** |
| 198 | HS102438 | CP025633.1 | I-E* | 2 | 35 | **+** |
| 199 | LS355 | CP025641.1 | I-E* | 1 | 22 | **+** |
| 200 | LS358 | CP025629.1 | 0 | 0 |  | ˗ |
| 201 | 207M1D0-sc-2013-04-03T11:21:06Z-1606409 | LT216436.1 | 0 | 4 |  | ˗ |
| 202 | FDAARGOS_440 | CP023919.1 | 0 | 3 |  | ˗ |
| 203 | FDAARGOS_436 | CP023907.1 | 0 | 4 |  | ˗ |
| 204 | FDAARGOS_447 | CP023949.1 | 0 | 4 |  | ˗ |
| 205 | FDAARGOS_442 | CP023925.1 | I-E | 4 | 43 | ˗ |
| 206 | FDAARGOS_443 | CP023933.1 | 0 | 5 |  | ˗ |
| 207 | KpvK54 | CP023134.2 | 0 | 2 |  | ˗ |
| 208 | 002SK2 | CP025515.1 | I-E | 2 | 34 | ˗ |
| 209 | yzusk-4 | CP011421.1 | 0 | 0 |  | ˗ |
| 210 | KP8 | CP025089.1 | I-E | 0 | 61 | ˗ |
| 211 | KP11 | CP025092.1 | I-E | 0 | 30 | ˗ |
| 212 | DT1 | CP019077.1 | I-E | 1 | 24 | ˗ |
| 213 | DT12 | CP019079.1 | I-E | 1 | 24 | ˗ |
| 214 | LS356 | CP025636.1 | I-E | 2 | 61 | **+** |
| 215 | LS359 | CP025630.1 | I-E | 0 | 11 | ˗ |
| 216 | Kp81 | CP025816.1 | 0 | 2 |  | ˗ |
| 217 | RYC492 | APGM01000001.1 | I-E | 0 | 11 | ˗ |

^a^ +, presence; -, absence
